# Reiterative use of FGF signaling in mesoderm development during embryogenesis and metamorphosis in the hemichordate *Ptychodera flava*

**DOI:** 10.1186/s12862-018-1235-9

**Published:** 2018-08-03

**Authors:** Tzu-Pei Fan, Hsiu-Chi Ting, Jr-Kai Yu, Yi-Hsien Su

**Affiliations:** 1 Molecular and Biological Agricultural Sciences Program, Taiwan International Graduate Program, National Chung Hsing University and Academia Sinica, Taipei, 11529 Taiwan; 2 Institute of Cellular and Organismic Biology, Academia Sinica, 128 Academia Rd., Sec. 2, Nankang, Taipei, 11529 Taiwan; 3 Graduate Institute of Biotechnology, National Chung Hsing University, Taichung, 40227 Taiwan; 4 Biotechnology Center, National Chung Hsing University, Taichung, 40227 Taiwan

**Keywords:** FGF, Mesoderm, *Ptychodera flava*, Hemichordate, Metamorphosis

## Abstract

**Background:**

Mesoderm is generally considered to be a germ layer that is unique to Bilateria, and it develops into diverse tissues, including muscle, and in the case of vertebrates, the skeleton and notochord. Studies on various deuterostome animals have demonstrated that fibroblast growth factor (FGF) signaling is required for the formation of many mesodermal structures, such as vertebrate somites, from which muscles are differentiated, and muscles in sea urchin embryos, suggesting an ancient role of FGF signaling in muscle development. However, the formation of trunk muscles in invertebrate chordates is FGF-independent, leading to ambiguity about this ancient role in deuterostomes. To further understand the role of FGF signaling during deuterostome evolution, we investigated the development of mesodermal structures during embryogenesis and metamorphosis in *Ptychodera flava*, an indirect-developing hemichordate that has larval morphology similar to echinoderms and adult body features that are similar to chordates.

**Results:**

Here we show that genes encoding FGF ligands, FGF receptors and transcription factors that are known to be involved in mesoderm formation and myogenesis are expressed dynamically during embryogenesis and metamorphosis. FGF signaling at the early gastrula stage is required for the specification of the mesodermal cell fate in *P. flava*. The mesoderm cells are then differentiated stepwise into the hydroporic canal, the pharyngeal muscle and the muscle string; formation of the last two muscular structures are controlled by FGF signaling. Moreover, augmentation of FGF signaling during metamorphosis accelerated the process, facilitating the transformation from cilia-driven swimming larvae into muscle-driven worm-like juveniles.

**Conclusions:**

Our data show that FGF signaling is required for mesoderm induction and myogenesis in the *P. flava* embryo, and it is reiteratively used for the morphological transition during metamorphosis. The dependence of muscle development on FGF signaling in both planktonic larvae and sand-burrowing worms supports its ancestral role in deuterostomes.

**Electronic supplementary material:**

The online version of this article (10.1186/s12862-018-1235-9) contains supplementary material, which is available to authorized users.

## Background

Mesoderm is a unique feature of bilaterians and contributes to the increased complexity of body structures. During vertebrate development, mesoderm is subdivided into several regions, including axial, paraxial, intermediate and lateral plate mesoderm. Each of these regions gives rise to different tissues. For example, axial mesoderm forms the notochord, while paraxial mesoderm forms somites, which further differentiate into muscle and many types of connective tissue [1–5]. In amphioxus, a basal chordate, the mesoderm also gives rise to notochord and somites. Ascidians, the sister group of the vertebrates, lack somites and their muscles and notochord are derived from mesodermal cells. The mesodermal origin of the muscle is well recognized, yet our understanding of the molecular mechanisms that control myogenesis in various animals is only recently emerging.

Studies in several vertebrate models have elucidated critical roles for fibroblast growth factor (FGF) signaling during mesoderm differentiation. For example, in *Xenopus*, FGF signaling is required for the development of axial and paraxial mesoderm. Disruption of FGF signaling by expressing a dominant negative form of the FGF receptor resulted in the loss of both notochord and somites [6–8]. In zebrafish, suppression of the FGF signal significantly reduces mesoderm formation, leading to the complete absence of a trunk and tail and the loss of *no tail* expression, which marks the dorsal axial mesoderm (putative notochord) [9–11]. On the basis of studies such as these, FGF signaling appears to play a conserved role in specifying notochord and somites during vertebrate development.

In order to decipher the ancestral role of FGF signaling in chordate mesoderm development, studies have also been carried out on invertebrate chordates, including amphioxus and ascidians. Intriguingly, FGF signaling was reported to only be required for the formation of anterior somites in amphioxus [12]. In ascidians, although FGF signaling is essential for the formation of notochord and secondary muscle, which is a small portion of the body musculature, differentiation of the trunk muscle is FGF-independent [13, 14]. Thus, the requirement of FGF signaling in notochord development appears to be evolutionarily conserved, at least in vertebrates and ascidians, while the molecular mechanisms that regulate myogenesis may have been altered during chordate evolution. In addition to the studies in chordates, the roles of FGF signaling in mesoderm development have also been studied in several model organisms. For example, in *Drosophila melanogaster*, the FGF receptor (*heartless*) null mutant embryos specified their primary mesoderm normally, but these tissues failed to go through its usual dorsolateral migration and thus resulted in the malformation of the body wall muscles [15, 16]. In *Caenorhabditis elegans*, mutations in FGF ligand *egl-17* and receptor *egl-15* affect the specification and migration of the larval sex myoblasts, a small subset of body muscle structures [17–20]. Because FGF regulation in myogenesis is inconsistent among different animals, whether this function represents an ancestral character remains unclear.

To gain insight into the ancestral function of FGF signaling for mesoderm, it is important to investigate Ambulacraria, which includes both echinoderms and hemichordates and is the closest group to the chordates [21–23]. In contrast to the extensive body of knowledge about the molecular details of mesoderm development in chordates, limited molecular analyses have been performed on ambulacrarians. Studies in sea urchin embryos have revealed that FGF signaling plays a central role in myogenesis and skeletal morphogenesis [24, 25]. In *Saccoglossus kowalevskii*, a direct-developing hemichordate acorn worm, it has been shown that inhibition of FGF signaling, by either downregulating *fgfr-b* or *fgf8/17/18*, resulted in the loss of the mesoderm and reduced the expression of several mesodermal and muscle marker genes [26]. Therefore, FGF signaling is clearly required for mesoderm induction in *S. kowalevskii*. However, it is unclear whether myogenesis is directly regulated by FGF signaling after mesoderm induction has completed. In the indirect-developing acorn worm, *Ptychodera flava*, it has been shown that U0126, an inhibitor against mitogen-activated protein kinase kinase (MEK), blocks mesoderm formation [27], although the identity of the upstream signaling molecule was not reported. Therefore, the potential involvement of FGF signaling in hemichordates during the process of mesoderm differentiation into muscles remains to be elucidated.

In this study, we investigated the roles of FGF signaling in mesoderm development during embryogenesis and metamorphosis of *P. flava*. Unlike the direct-developing *S. kowalevskii*, which grows directly into a vermiform body [28, 29], *P. flava* develops into a swimming larva before metamorphosing into a worm-like adult body plan [30–33]. The swimming tornaria larva is propelled by cilia and shares several morphological features with echinoderm larvae [34, 35]. During metamorphosis, *P. flava* changes its body form and lifestyle dramatically, becoming a bottom-burrower with a muscular vermiform body [30, 31]. Here, we show that FGF signaling regulates diverse transcription factors to induce mesoderm specification and muscle differentiation in *P. flava* during different embryonic time windows. We also show that, in addition to environmental stimulations, FGF signaling is able to accelerate the progression of metamorphosis. These data suggest an ancient role for FGF signaling in muscle development in the common ancestor of deuterostomes.

## Methods

### Embryonic culture and metamorphosis

Mature *P. flava* were collected from Chito Bay, Penghu Islands, Taiwan, as previously described [30]. Spawning and embryo cultivation were conducted according to methods described previously [30, 33, 36] with modifications. In short, worms were kept in the dark at 22 °C overnight before shifting them to a 30 °C incubator to stimulate spawning. The seawater was changed with the pre-warmed seawater (30 °C) every 15 min until spawning occurred. Embryos were cultured in 0.22 μm-filtered seawater (FSW) containing penicillin and streptomycin, 50 mg/L each (antibiotic-FSW) at 23 °C. After hatching, the larvae were raised until metamorphosis based on published methods [30, 31] with some modifications. In brief, the tornaria larvae were kept at room temperature in a density of 1 larva/ml and agitated by a bubbling system. FSW was changed three times per week. Larvae were fed with *Rhodomonas lens* at 5000 cells/ml. Under this condition, the cultures reached the Spengel stage within 3 months. Advanced Spengel larvae, with fully developed protocoel, mesocoels and metacoels were picked for metamorphosis experiments. Sea sand was collected from the adult habitat and used to induce metamorphosis. The sand was first filtered through a 300 μm mesh, washed with FSW three times, and soaked in antibiotic-FSW overnight at room temperature. After removing the antibiotic-FSW and washing three times with FSW, the sand was ready to be used for metamorphosis experiments. For the experiments using sterilized sea sand, sand was washed with reverse osmosis-purified water three times, autoclaved, and then washed three times with FSW.

### Pharmacological and protein treatments

Embryos were treated with inhibitors or recombinant proteins after fertilization or at the indicated developmental stages. Treatments were maintained until fixation. Experiments performed at specific embryonic stages were repeated at least three times using different batches of *P. flava* that were derived from two or three separate breeding seasons. For each trial of the metamorphosis experiments, 10–15 Spengel larvae per condition were cultured in the presence of sand with or without the inhibitors or proteins. Each experiment was carried out over at least three replicate trials, unless otherwise indicated. The inhibitors and recombinant proteins used in this work include: SU5402 (Calbiochem, #572630), PD173074 (SIGMA, #P2499), U0126 (Calbiochem, #662005) and human basic-FGF (bFGF, SIGMA, #F0291). Inhibitors and recombinant protein were dissolved in DMSO or 0.1% BSA (v/v, in phosphate buffer saline), respectively. The solvents were used to treat experimental control groups.

### Whole-mount in situ hybridization and phalloidin staining

Digoxigenin-labeled RNA antisense probes were synthesized from cDNA clones using DIG RNA Labeling mix (Roche) and T7 or SP6 RNA polymerase (Promega). Fixation, dehydration and in situ hybridization were performed as described [36], and BM-purple (Roche) was used as a substrate for the alkaline phosphatase (AP). The Spengel larvae, Agassiz larvae and juveniles were relaxed in FSW containing 0.25 M magnesium chloride for 2 min before fixing in 4% paraformaldehyde. For phalloidin staining, samples were stored in phosphate-buffered saline containing 0.1% tween-20 (PBST) and 0.1% sodium azide (NaN_3_) after fixation. Following three washes with PBST, the samples were incubated in PBST with 0.1% Triton X-100 for 20 min, and then transferred to the blocking solution (PBST with 1% BSA) for at least 1 h at room temperature. Alexa Fluor 488 phalloidin (Invitrogen, #A12379, 1:50 dilution in the blocking solution) staining was performed at 4 °C overnight. Counterstaining was conducted using Hoechst 33342 (Invitrogen, #H1399, 1:1000 dilution in PBST) for 1 h. Extensive washes were performed between every step. Specimens were mounted in PBST containing 70% glycerol and 0.1% NaN_3_, and imaged with either a Zeiss (Axio Imager A2) microscope (for embryos) or Leica (Z16APO) microscope (for specimens larger than 1 mm). All phalloidin stained samples were imaged on a Leica (TCS-SP5) confocal microscope.

### Molecular cloning

All genes investigated in this study were cloned by methods described previously [37], and the sequences have been deposited in the GenBank (see Additional file 1: Table S1 for the accession numbers). In brief, cDNA sequences of homologous genes from *S. kowalevskii* were retrieved from GenBank and used as queries to search the *P. flava* embryonic transcriptome database [38]. Specific primers were designed for PCR (see Additional file 1: Table S2). RNA Ligase Mediated Rapid Amplification of cDNA Ends (RLM-RACE, Ambion) was used to obtain the full-length sequences. Total RNA was extracted using TRIzol reagent (Invitrogen) from several developmental stages, including unfertilized eggs, embryos at 12, 17, 25, 32, 43 and 64 hpf, and the Spengel, Agassiz and juvenile stages. The extracted RNA was further purified using an RNeasy Micro kit (Qiagen, Chatsworth, CA, USA). For cloning, the purified RNAs from each stage were reverse transcribed separately to obtain cDNA.

## Results

### Dynamic expression patterns of FGF ligands and receptors during *P. flava* embryogenesis

**Fig. 1:**
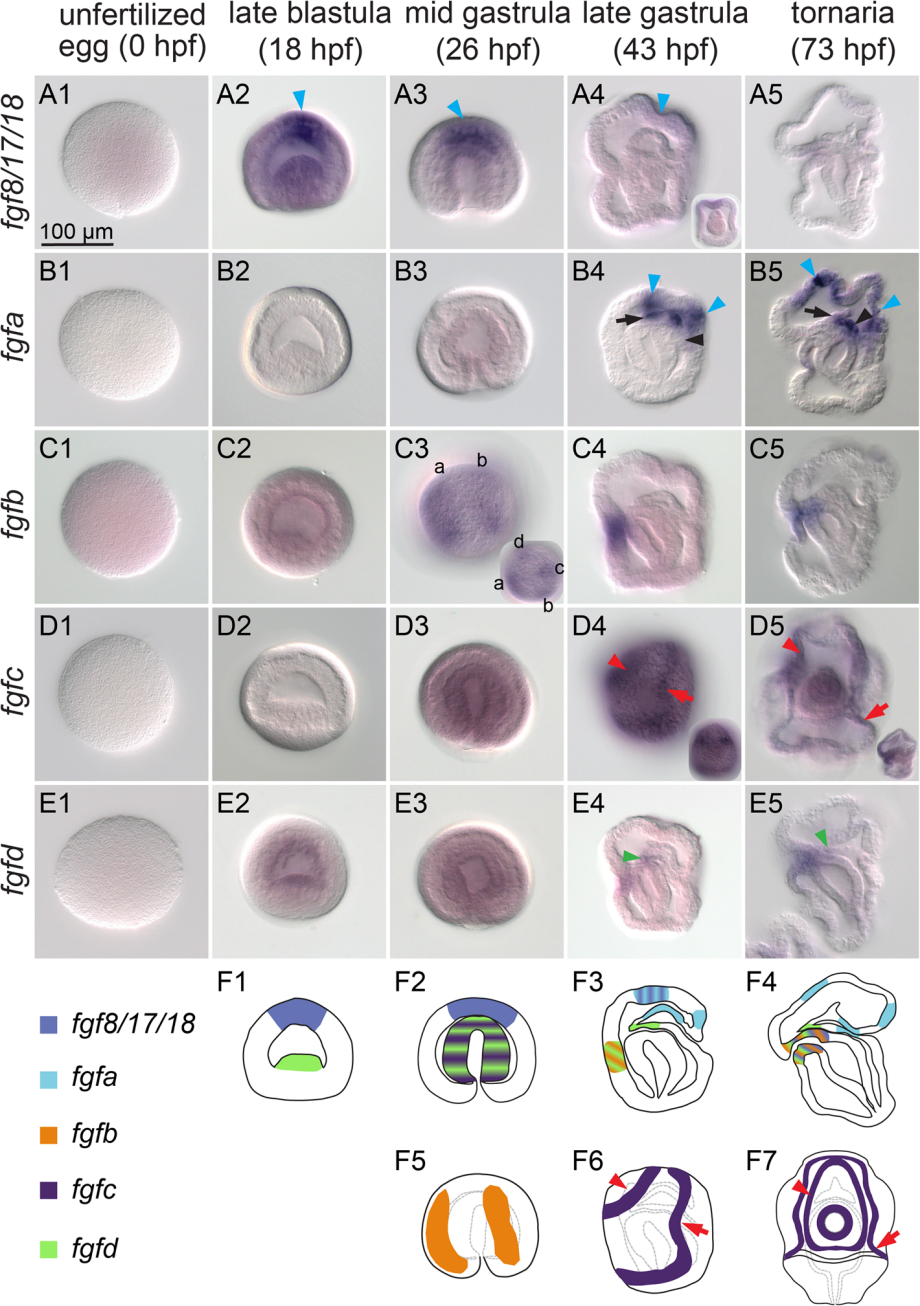
FGF ligand genes are dynamically expressed during *P. flava* embryogenesis. In situ hybridization of *fgf8/17/18* (A1-A5), *fgfa* (B1-B5), *fgfb* (C1-C5), *fgfc* (D1-D5) and *fgfd* (E1-E5) at different embryonic stages. All embryos are shown from a lateral view and oriented with the mouth to the left, except for panel D5. The embryo in D5 is shown from the ventral side. Embryos shown in C3 and D4 were photographed with the focal plane on the ectoderm. Inserts in A4, C3, D4 and D5 are shown from the ventral side, the apical surface, the ventral surface, and the lateral side, respectively. The expression patterns for all ligands are schematically summarized in F1-F7. Each stage is shown and different genes are indicated with different colors. The blue arrowheads in panels A2-A4 and B4-B5 indicate ectodermal expression. Black arrows in B4-B5 indicate the animal part of the protocoel, and the hydroporic canal is indicated by a black arrowhead. The four vertical stripes of *fgfb* expression are labeled with lowercase letters (a, b, c, and d) in the C3 insert. Two of the four stripes are circled by white dashed lines in C3 and labeled with lowercase letters (a and b), corresponding to the labels in the insert. Red arrows and arrowheads in panels D4-D5 and F6-F7 indicate the expression of *fgfc* in preoral and postoral ciliary bands. Green arrowheads in E4-E5 indicate the expression of *fgfd* in the ventral tip of the mesoderm. All panels are shown in the same scale, according to the scale bar in A1

The mesoderm of *P. flava* differentiates into several different structures during embryogenesis and metamorphosis (Additional file 2: Figure S1). To investigate the potential roles of FGF signaling during embryogenesis, we first characterized the expression patterns of genes encoding FGF ligands and receptors at several developmental stages, ranging from the unfertilized egg [0 h post fertilization (hpf)] to the tornaria stage (73 hpf). Among the five FGF ligands (*fgf8/17/18*, *fgfa*, *fgfb*, *fgfc* and *fgfd*) and three FGF receptors (*fgfra1*, *fgfra2* and *fgfrb*) identified previously [37], none were detected in the unfertilized egg (Fig. 1 A1-E1), indicating a lack of maternal FGF signaling. We found that *Fgf8/17/18* was the earliest *fgf* gene to be activated specifically in the apical ectoderm at the late blastula stage (Fig. 1 A2). The expression of *fgf8/17/18* remained in the apical ectoderm in the mid gastrula (Fig. 1 A3), but was diminished at the late gastrula and the tornaria stages (Fig. 1 A4-5). Transcription of *fgfa* was detected initially at the late gastrula stage in three domains, including the apical ectoderm, the animal dorsal ectoderm and the animal part of the protocoel (Fig. 1 B4). In the tornaria, *fgfa* expression remained broadly detectable in the apical ectoderm and animal dorsal ectoderm, but the expression in the protocoel became restricted to the hydroporic canal (Fig. 1 B5). The expression of *fgfb* was first detected during gastrulation in the ectoderm as four stripes along the animal-vegetal axis and these stripes were not interconnected (Fig. 1 C3). After gastrulation, *fgfb* expression was restricted to the stomodeum region in the ventral ectoderm (Fig. 1 C4-5). *Fgfc* transcripts were initially detected in the archenteron of the mid gastrula (Fig. 1 D3). At the late gastrula stage, expression was observed as two ectodermal rings (Fig. 1 D4), which were coincident with the positions of the developing ciliary bands. At the tornaria stage, *fgfc* was expressed in both the preoral and the postoral ciliary bands, in addition to its expression in the stomodeum and the pharynx (Fig. 1 D5 and F7). Expression of *fgfd* was observed in the endomesoderm at the late blastula and the mid gastrula stages (Fig. 1 E2-3). Two other expression domains appeared at the late gastrula and tornaria stages in the ventral ectoderm and the ventral tip of the mesoderm (Fig. 1E4-5 ). The expression of *fgfd* in the mouth/pharynx region was less pronounced than *fgfb* and *fgfc*, with a smaller domain and lower labeling intensity*.*

**Fig. 2:**
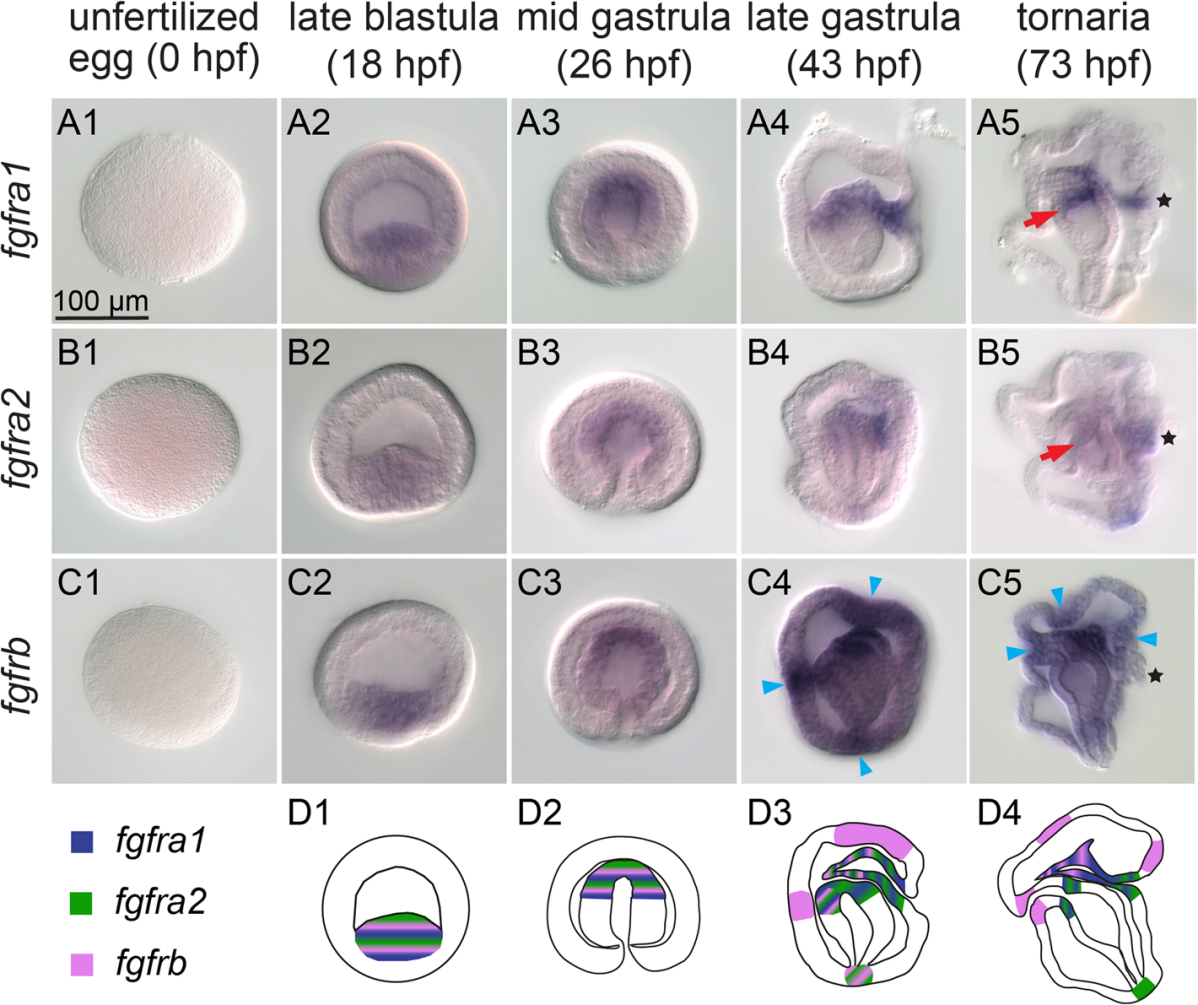
FGF receptor genes are expressed in mesodermal cells. In situ hybridization of *fgfra1* (A1-A5), *fgfra2* (B1-B5) and *fgfrb* (C1-C5) at different embryonic stages. The embryos are shown from a lateral view and oriented with the mouth to the left. Red arrows in A5 and B5 indicate the expression in the sphincter. Blue arrows in C4-C5 indicate ectodermal *fgfrb* expression domains. Black asterisks mark the hydropore in A5-C5. The expression patterns of the FGF receptor genes in the developing mesodermal cells and other embryonic territories are summarized schematically at the corresponding stages in D1-D4. All panels are shown at the same scale, according to the scale bar in A1

The expression of all three FGF receptors was observed in the endomesoderm at the blastula stage (Fig. 2 A2-C2) and then became restricted to the tip of the archenteron during gastrulation (Fig. 2 A3-C3). At the late gastrula stage, *fgfra1* and *fgfra2* expression remained in most of the mesodermal cells (Fig. 2 A4-B4), while the *fgfrb* transcript level was higher in the ventral part of the mesoderm but lower in the developing hydroporic canal (Fig. 2 C4). In addition, all three FGF receptor genes were expressed in the anterior archenteron, where it later bends toward the ventral ectoderm to form the mouth (Fig. 2 A4-C4). At the tornaria stage, expression of *fgfra1* and *fgfrb* remained in the mesoderm (Fig. 2 A5 and C5), whereas *fgfra2* expression disappeared in most mesoderm-derived structures, except for the cells near the hydropore (Fig. 2 B5). Expression of *fgfra1* and *fgfra2* in the anterior archenteron was restricted to the sphincter that connects the esophagus and the stomach (Fig. 2 A5 and B5), while the expression of *fgfrb* was detected at the mouth region (Fig. 2 C5). *Fgfrb* transcripts were also detected in several ectodermal patches, including the apical, ventral, dorsal and blastopore regions, at both the late gastrula and tornaria stages (Fig. 2 C4-C5). Our in situ hybridization analyses revealed that genes encoding FGF ligands and receptors are expressed dynamically throughout embryogenesis (Figs. 1 F1-7 and 2 D1-4). Although in situ hybridization analysis is not absolutely quantitative, the changes of the expression levels observed at different stages are mostly consistent with the temporal expression profiles analyzed previously by using quantitative PCR (QPCR) [37]. These data suggest that *fgf8/17/18*, *fgfb* and *fgfd* participate in the early development before or during early gastrulation, while *fgfa* and *fgfc* are more important at the late gastrula and the tornaria stages. Furthermore, the expression of FGF receptors in mesodermal cells strongly suggests that FGF signaling is involved in the developmental processes of the mesoderm.

### FGF signaling is essential for mesoderm induction in *P. flava*

**Fig. 3:**
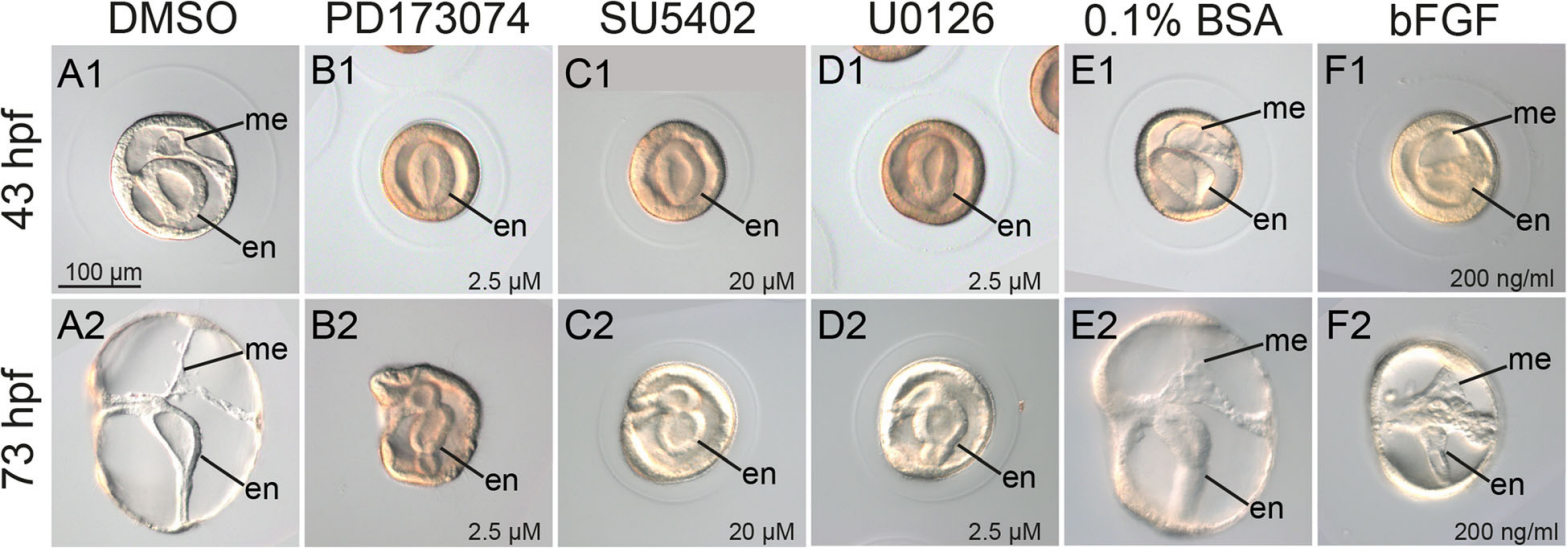
FGF signaling is required for mesoderm induction. Phenotypes of embryos at 43 hpf (A1-F1) and 73 hpf (A2-F2) after treatment with FGF signaling inhibitors (B1-D2) or bFGF protein (F1-F2) upon fertilization. Control embryos were treated with DMSO or 0.1% BSA. The concentrations of each drug or protein are indicated in each panel. All embryos are shown from a lateral view with mouth on the left. All panels have the same scale, with the scale bar marked in A1. Abbreviations: me: mesoderm; en: endoderm

To examine the role of FGF signaling in specifying the mesoderm of *P. flava*, we inhibited FGF signaling after fertilization by using either FGF receptor inhibitors (PD173074 or SU5402) or an inhibitor (U0126) against MEK, a downstream kinase of the FGF signaling. We also used exogenous recombinant FGF protein (bFGF) to enhance FGF signaling in the embryos. Two concentrations of each inhibitor and protein were used to determine optimal conditions for the following experiments (effects of higher concentrations are shown in Fig. 3, and lower concentrations are shown in Additional file 3: Figure S2). Control embryos showed normal mesoderm development at both 43 hpf (late gastrula) and 73 hpf (tornaria) (Fig. 3 A1-2; E1-2). Embryos treated with any single FGF receptor or MEK inhibitor lack mesoderm completely at both developmental stages and became smaller at the tornaria stage, although gastrulation occurred normally and the endoderm differentiated into a tripartite gut (Fig. 3 B1-D2). On the other hand, mesodermal tissues were substanially expanded when FGF signaling was elevated by the addition of bFGF protein to the embryo culture (Fig. 3 F1-2). The drug effects were highly penetrant (> 99% at the optimal concentrations). From these observations, we conclude that mesoderm induction is controlled by FGF signaling in *P. flava*, and the signaling may be transduced through the MEK pathway.

### FGF signaling is required stepwise for the formation of the mesoderm-derived structures

**Fig. 4:**
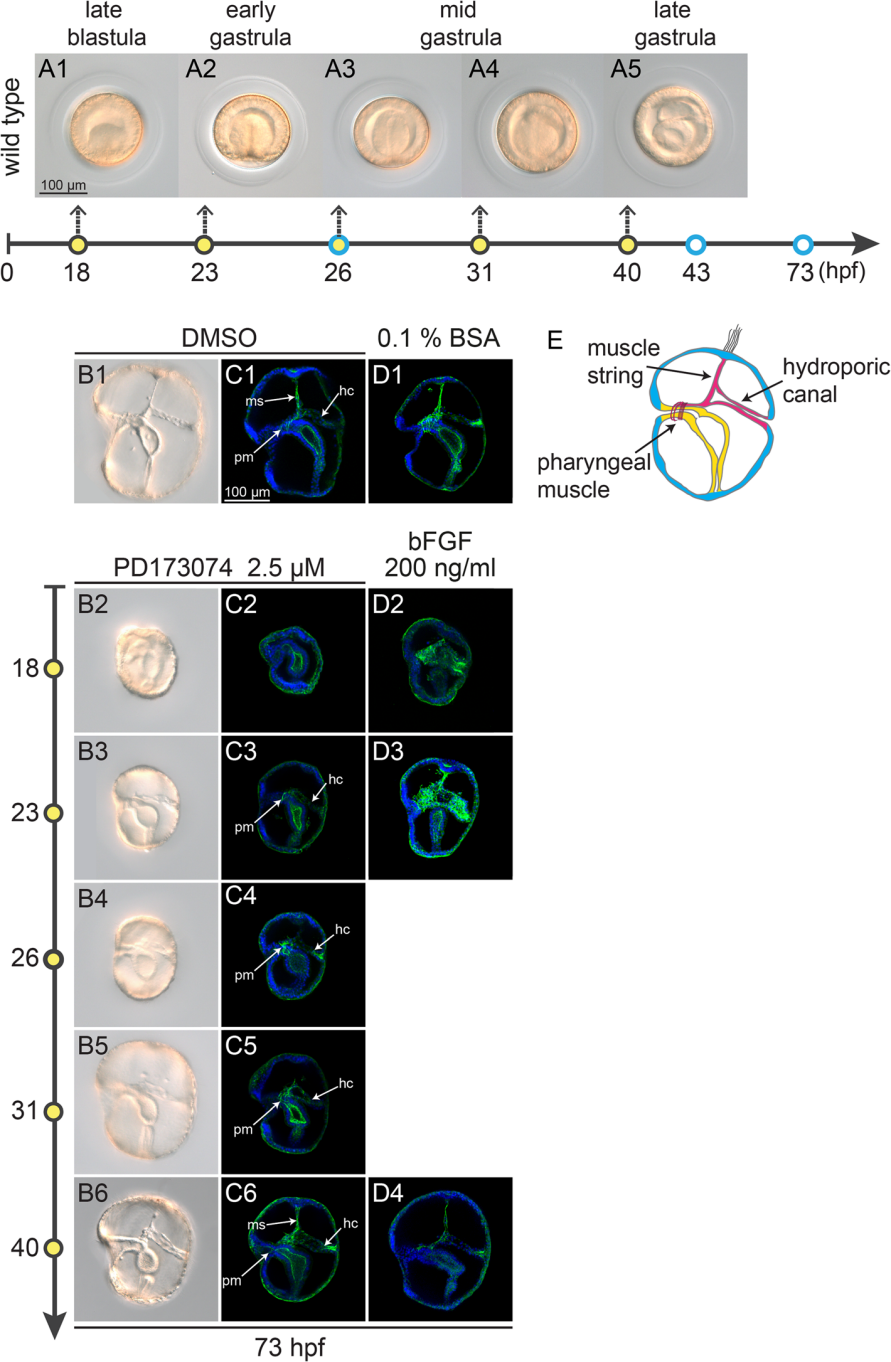
FGF signaling stepwise regulates the formation of the mesoderm-derived structures. (A1-A5) Panels show the morphology of wild type embryos at the time points indicated by the solid yellow circles. Treatments were performed at the same time points, and embryos were observed at the time points indicated by the blue circles. (B1-B6) Phenotypes of the tornaria larvae treated with DMSO (B1) or PD173074. PD173074 was applied at different stages of development, indicated by the yellow circles on the left (B2-B6). (C1-D4) The tornaria larvae (73 hpf) treated with PD173074 (C2-C6) or bFGF (D2-D4) at different developmental stages were stained with Phalloidin (green). The penetrance of the drug effects was high (> 99%) when treated at either 18 or 40 hpf. When treated at 23, 26 or 31 hpf, ~ 70% of the larvae did not exhibit a muscle string. The efficiency of bFGF protein was consistently high (> 99%) for all the treatments. The larvae were counterstained with Hoechst 33,342 for nuclei (blue). **e** Illustration of a tornaria larva with mesodermal structures in red. The pharyngeal muscle, the muscle string and the hydroporic canal are indicated. The scale bar in A1 is shown for panels A1-B6, and the scale bar in C1 is shown for panels C1-D4. Abbreviations: ms, muscle string; pm, pharyngeal muscle; hc, hydroporic canal

*P. flava* embryos normally develop several mesoderm-derived structures, including the hydroporic canal, pharyngeal muscle and the muscle string (Fig. 4e). To investigate whether FGF signaling is required for the proper formation of these structures, we treated embryos with FGF receptor or MEK inhibitors at different stages (Fig. 4 A1-5). Similar to the treatments performed after fertilization (Fig. 3 B1-2), inhibition of FGF signaling by PD173074 at 18 hpf abolished all mesoderm (Fig. 4 B2, C2, and Additional file 4: Figure S3B1), indicating that the mesodermal cell fate has not been determined at the blastula stage. Mesoderm induction was no longer affected when FGF signaling was blocked at or beyond the early gastrula stage (23 hpf) (Fig. 4 B3-6, and Additional file 4: Figure S3B2–4), suggesting that mesoderm is specified during early gastrulation, between 18 and 23 hpf. In embryos treated with inhibitors at 23 hpf, the hydroporic canal developed normally, but the number of the pharyngeal muscle fibers was clearly decreased and the muscle string was completely absent (Fig. 4 B3 and C3). Treatments performed at 26 hpf and 31 hpf showed a similar phenotype except that the number of muscle fibers around the pharynx was less affected (Fig. 4 B4-5 and C4-5). The three mesodermal structures appeared normally in embryos treated at the late gastrula stage (40 hpf) (Fig. 4 B6 and C6). Similar results were observed in embryos treated with SU5402 and U0126 (Additional file 4: Figure S3), although SU5402 caused a more severe phenotype in which the hydroporic canal was unable to extend to the dorsal ectoderm when the treatments were performed before 31 hpf (Additional file 4: Figure S3E2–4 and 3F2–4). On the other hand, elevating FGF signal with exogenous bFGF protein at 18 or 23 hpf caused enlargement of the hydroporic canal and an increased number of muscle fibers (Fig. 4 D2-3). Notably, although inhibition of FGF signaling showed no effect on gut development, elevating FGF signaling at 18 and 23 hpf affected pharynx development. These sequential developmental effects of modulating FGF signaling show that the mesoderm-derived structures are specified in a stepwise manner. The order of specification begins from the hydroporic canal, followed by the pharyngeal muscle and the muscle string. Moreover, FGF signaling is required for the formation of the two muscular structures, the pharyngeal muscle and muscle string, but it is not required for formation of the hydroporic canal.

### FGF signaling activates the expression of mesodermal transcription factor genes

**Fig. 5:**
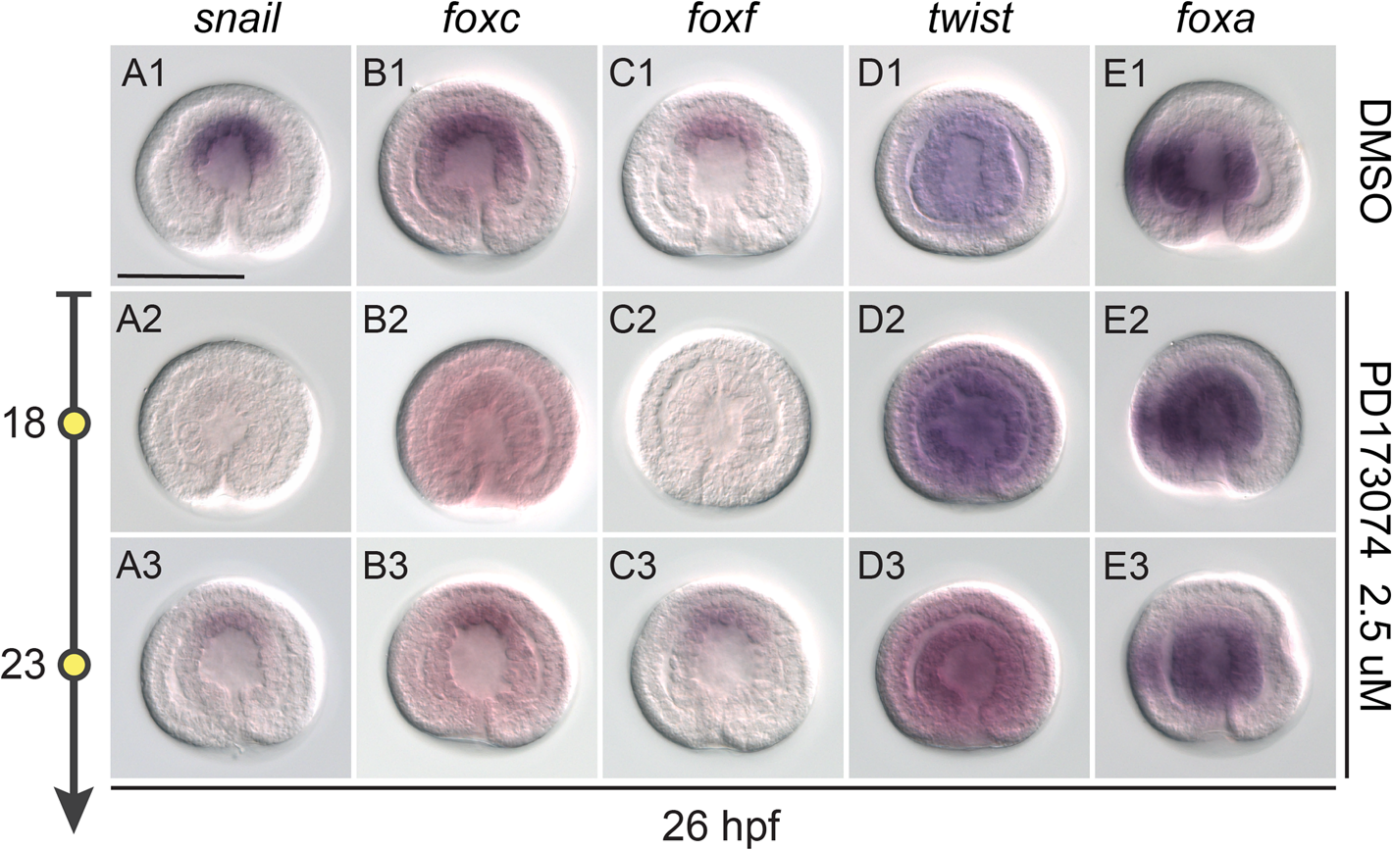
FGF signaling regulates the expression of the mesodermal transcription factor genes in the presumptive mesoderm. In situ hybridization for *snail* (A1-A3), *foxc* (B1-B3), *foxf* (C1-C3), *twist* (D1-D3) and *foxa* (E1-E3) in 26 hpf embryos treated with DMSO (A1-E1) or 2.5 μM PD173074 at 18 hpf (A2-E2) or 23 hpf (A3-E3). All panels are shown in the same scale, according to the scale bar (100 μm) in A1

To further understand the potential molecular mechanisms underlying mesoderm induction in *P. flava*, we analyzed the expression of several genes that encode transcription factors known to participate in mesoderm development across several bilaterian phyla. These genes include *twist* [39], *snail* [26, 40–46], *foxc* and *foxf* [24, 47–50]. Among the genes we studied, *snail* and *foxc* were the first to exhibit specific expression in the presumptive endomesoderm at the blastula stage (Additional file 5: Figure S4A2 and S4B2). During gastrulation, *snail*, *foxc* and *foxf* were expressed specifically at the tip of the archenteron, where the presumptive mesodermal cells are located (Additional file 5: Figure S4A3–5, S4B3–5, and S4C4–5). Inhibition of FGF signaling by PD173074 at 18 hpf abolished the expression of *snail*, *foxc* and *foxf* (Fig. 5 A2-C2) in the presumptive mesoderm, preceding a complete loss of mesodermal cells in later stages (Fig. 4 B2 and C2). When signaling was inhibited at 23 hpf, the expression levels of these three genes were decreased, but still detectable (Fig. 5 A3-C3). This result may explain why we observed a phenotype in which some mesoderm-derived structures, the hydroporic canal and some pharyngeal muscles, still formed under the same treatment schedule (Fig. 4 B3-C3). The expression of *twist* was not specific to the mesoderm, and although the background was generally high, we consistently detected a weak signal in the entire archenteron (Additional file 5: Figure S4D3). *Twist* expression was upregulated when FGF signaling was inhibited (Fig. 5 D2-3). We also analyzed the expression of *foxa*, an endodermal marker [51]. In the control embryo, *foxa* was expressed in the presumptive stomodeal region and the endodermal part of the archenteron, but not in the most anterior part of the archenteron, where the presumptive mesoderm is located (Fig. 5 E1). When FGF signaling was blocked at 18 hpf, the *foxa* expression domain expanded to the anterior part of the archenteron (Fig. 5 E2), suggesting that the invagination tissue became entirely endodermal fate. This data further confirms that mesodermal tissue is completely eliminated when FGF signaling is blocked at this stage. When FGF signaling is inhibited at 23 hpf, *foxa* expression remained in the endodermal part of the archenteron, consistent with the detectable expression of mesodermal markers (Fig. 5 A3-C3, E3). The increased transcript level of *twist* upon FGF signaling inhibition also implies that normally *twist* is negatively regulated by FGF signaling in the endoderm. These results suggest that FGF signaling induces the mesodermal cell fate through activation of *snail*, *foxc* and *foxf* expression.

### The regulatory states of the mesoderm progressively changed during embryogenesis

We then further examined the expression of the same four transcription factor genes in conjunction with other known myogenic genes in later stages of development. In these experiments, we tracked the differentiation of mesoderm into the three different structures, including the hydroporic canal, the pharyngeal muscle and the muscle string. In situ hybridization of the myogenic *myocardin* gene [52–55] showed high background and no specific expression pattern was recognizable in early embryonic stages (Additional file 5: Figure S4E1–5). At the late gastrula and tornaria stages, *myocardin* expression could be observed in restricted regions of the mesoderm (Additional file 5: Figure S4E6–7). In these later stages, the expression domains of *myocardin*, *twist*, *foxc* and *foxf* covered different regions of the developing mesoderm. At the late gastrula and tornaria stages, *snail* expression was diminished, while *twist* transcripts were detected in the ventral tip of the mesoderm; *foxc* and *myocardin* were also expressed in the ventral tip as well as the middle part of the mesoderm, but not in the hydroporic canal; *foxf* mRNA was detected in the entire mesoderm (Additional file 5: Figure S4A6-E7). The two type II *myosin heavy chain* (*MHC*) genes, striated muscle MHC (*stMHC*) and smooth muscle MHC (*smMHC*) (Additional file 6: Figure S5A; Additional file 7 for supplementary methods), displayed different expression patterns. st*MHC* was initially expressed in the ventral and middle regions of the mesoderm, and later it was expressed in the pharyngeal muscle and the muscle string of the tornaria larva (Additional file 6: Figure S5B1–2). sm*MHC* transcripts were not detected in the two muscular structures but were mainly observed in the dorsal ectoderm (Additional file 6: Figure S5C1–2). Notably, one of the earlier described FGF ligand genes, *fgfa*, was also specifically expressed in the middle part of the mesoderm, similar to *foxc*, *myocardin* and *foxf*, with later expression in the hydroporic canal (Fig. 1 A4-5). Together, these results show that the *P. flava* mesoderm is progressively compartmentalized to form ventral (*twist-*, *foxc-*, *myocardin-* and *foxf*-positive), middle (*foxc-*, *myocardin-*, *fgfa-* and *foxf*-positive) and dorsal (*foxf*-positive) domains at the late gastrula stage. These domains represent different regulatory states, which possibly contribute to the formation of the three mesodermal structures, the pharyngeal muscle, the muscle string and the hydroporic canal.

### Myogenic regulatory factors are controlled by FGF signaling

**Fig. 6:**
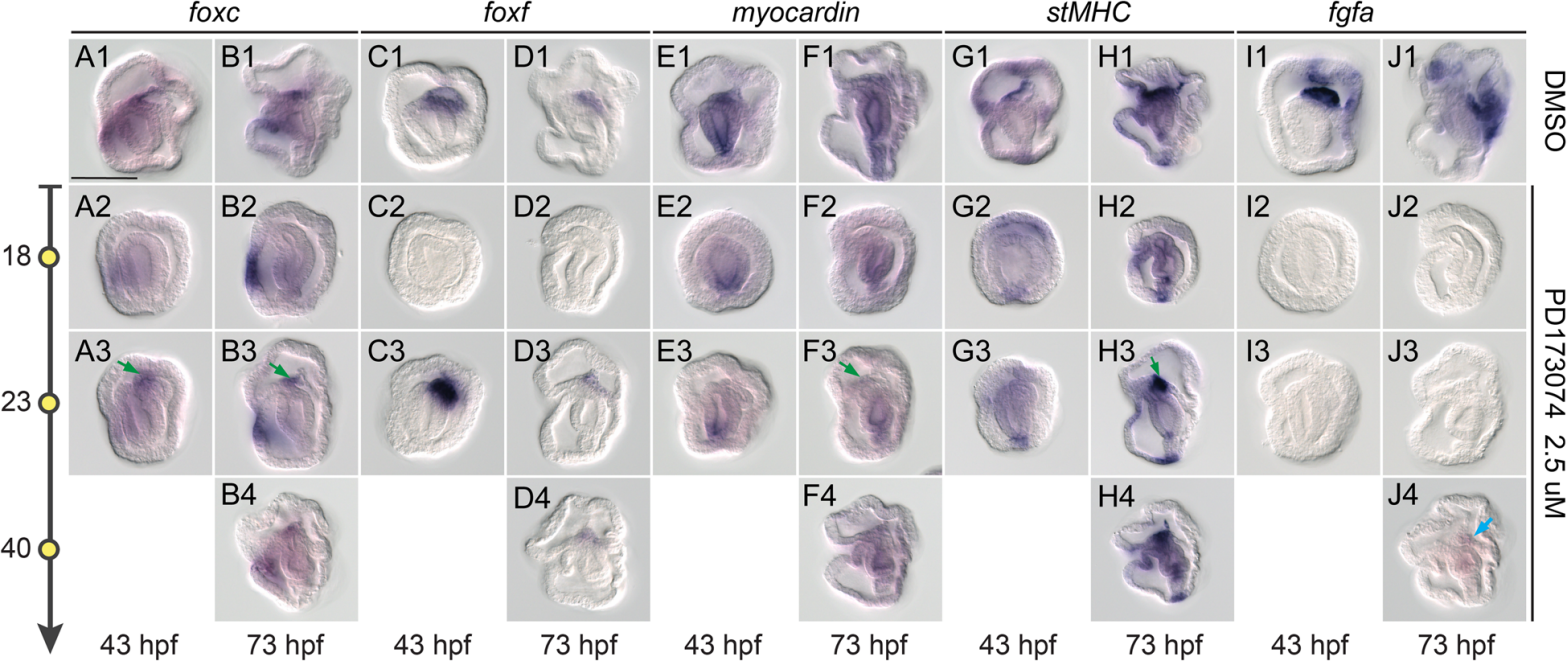
FGF signaling regulates the expression of the mesodermal and myogenic genes. Expression patterns of *foxc* (A1-B4), *foxf* (C1-D4), *myocardin* (E1-F4), st*MHC* (G1-H4) and *fgfa* (I1-J4) were analyzed in 43 hpf or 73 hpf embryos treated with DMSO or 2.5 μM PD173074 at various developmental stages (indicated by the yellow circles on the left). Embryos were observed from the lateral side with the mouth to the left. All panels are shown in the same scale, according to the scale bar (100 μm) in A1. Green arrows in A3, B3, F3 and H3 mark expression of the indicated gene at the ventral tip of the mesoderm/pharyngeal muscle. Blue arrow in J4 indicates the expression of *fgfa* in the hydroporic canal

When FGF signaling was blocked at 18 hpf, no mesoderm developed, and as such, no mesodermal gene expression could be observed at the later developmental times of 43 and 73 hpf (Fig. 6 A2-J2). When FGF signaling was blocked at 23 hpf, the expression domain of *foxc* shrank to the most ventral tip of the mesoderm (Fig. 6 A3-B3), while the mesodermal expression domains of *myocardin* and *stMHC* disappeared at 43 hpf (Fig. 6 E3, G3). Later, at 73 hpf, faint *myocardin* expression was detected, but it was only in the ventral tip of the mesoderm (Fig. 6 F3), while *stMHC* expression in the pharyngeal muscle was fully retained (Fig. 6 H3). In contrast, *foxf* expression was mostly unaffected when FGF signaling was blocked at either 23 or 40 hpf (Fig. 6 C3, D3-D4), coincident with normal development of the hydroporic canal under these conditions. Additionally, expression of *fgfa* was completely abolished when FGF signaling was blocked, suggesting the existence of a positive feedback loop between FGF signaling and *fgfa* expression (Fig. 6 I2-J3). When FGF inhibitor was administered at 40 hpf, most of the genes investigated retained expression, albeit at a lower level (Fig. 6 B4-J4). These results indicate that during gastrulation, continued FGF signaling is important for the formation of the pharyngeal muscle and the muscle string, probably via the maintenance of proper *foxc*, *myocardin*, st*MHC* and *fgfa* expression in the ventral and middle domains of the mesoderm. On the other hand, *foxf* expression is not controlled by FGF signaling after the mesoderm is specified. This result is consistent with the observation that the dorsal domain, which is marked by the expression of *foxf*, but not by other genes examined, still developed into the hydroporic canal when FGF signaling is blocked after mesoderm induction.

### Muscle fibers are extensively generated during metamorphosis

**Fig. 7:**
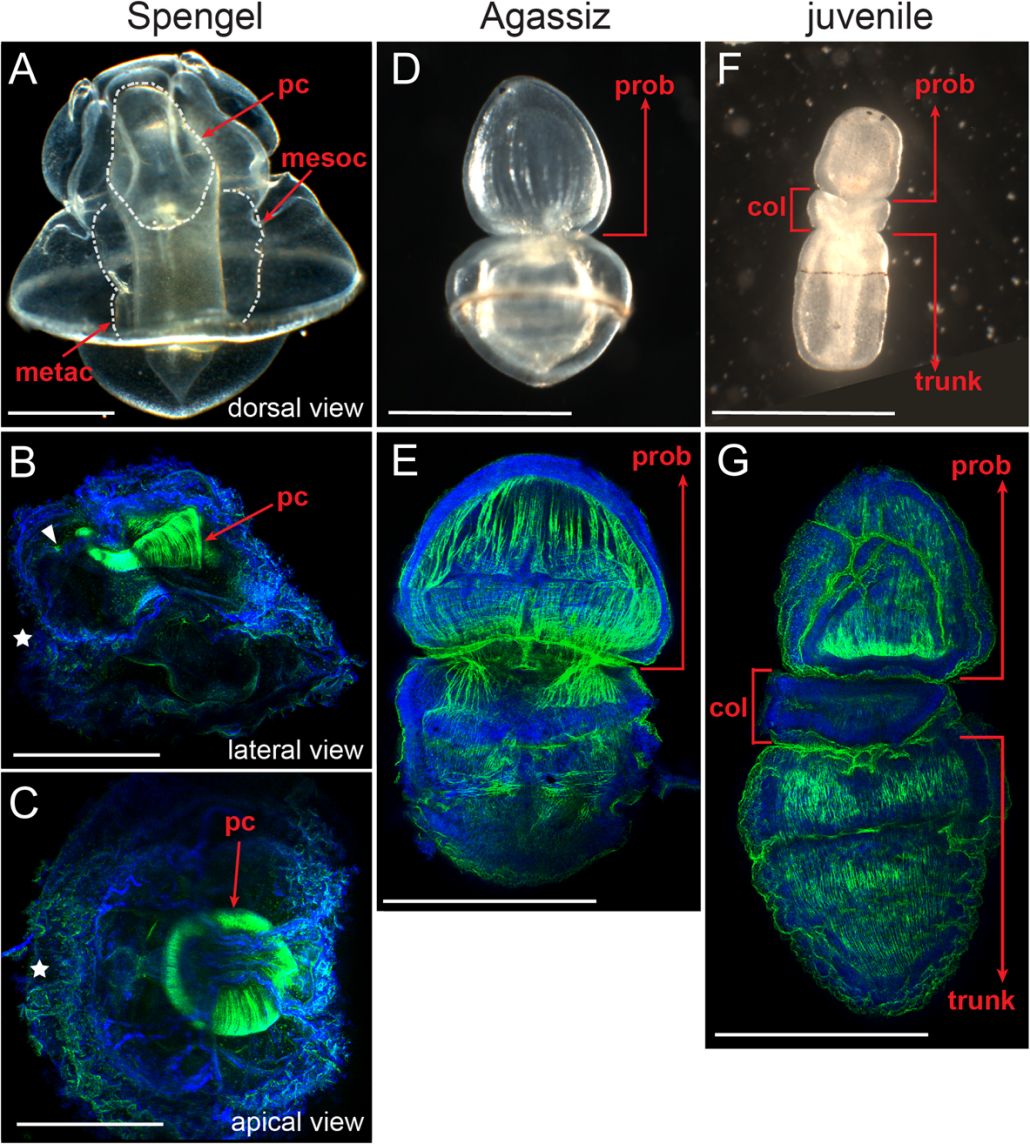
Muscle fibers are extensively generated during metamorphosis. Morphological changes during the *P. flava* transition from the Spengel (**a**-**c**) to the Agassiz (**d**-**e**) and then to the juvenile stage (**f**-**g**). Fully developed protocoel, mesocoel and metacoel are outlined by white dashed lines in panel **a**. Phalloidin staining (green) revealed the distribution of muscle fibers in the protocoel of the Spengel larva (**b** and **c**, viewed from the lateral and the apical side, respectively), and in the proboscis and trunk regions at the Agassiz (**e**) and the juvenile (**g**) stages. The asterisks in (**b**) and (**c**) indicate the position of the mouth. Nuclei were counterstained with Hoechst 33,342 (blue). Scale bar: 1 mm. Abbreviations: pc, protocoel; mesoc, mesocoel; metac, metacoel; prob., proboscis; col., collar

Indirect developing *P. flava* spend months as swimming larvae. The competent Spengel larvae then transform into Agassiz larvae and later metamorphose into juveniles with a vermiform body plan (Additional file 5: Figure S1E-H). Previous studies have shown that sand from the adult habitat is an important environmental factor that triggers this morphological transition [29–31, 56]. Because this dramatic morphological change from a cilia-driven plankton is also accompanied by a lifestyle change to a muscle-driven benthic worm, we endeavored to investigate muscle development during this transition. At the Spengel larval stage, most muscle fibers were observed surrounding the protocoel (Fig. 7a-c), and a small amount of muscle was found around the pharynx (Fig. 7b, white arrowhead). When the Spengel larva was cultured with sand, it transformed into the Agassiz stage within 1 day, and during this transformation, muscles were extensively generated in the enlarged protocoel (Fig. 7d-e), which later contributed to a highly muscular proboscis. Some muscle fibers also began to develop in the posterior region during the transition to the Agassiz stage (Fig. 7e). After metamorphosis, the muscle fibers were well developed in the proboscis and the trunk region of the juvenile worm (Fig. 7f-g). The mass production of muscles during metamorphosis reflects and correlates with the lifestyle transition from planktonic larvae to benthic worms.

### FGF signaling accelerates the progress of metamorphosis

**Fig. 8:**
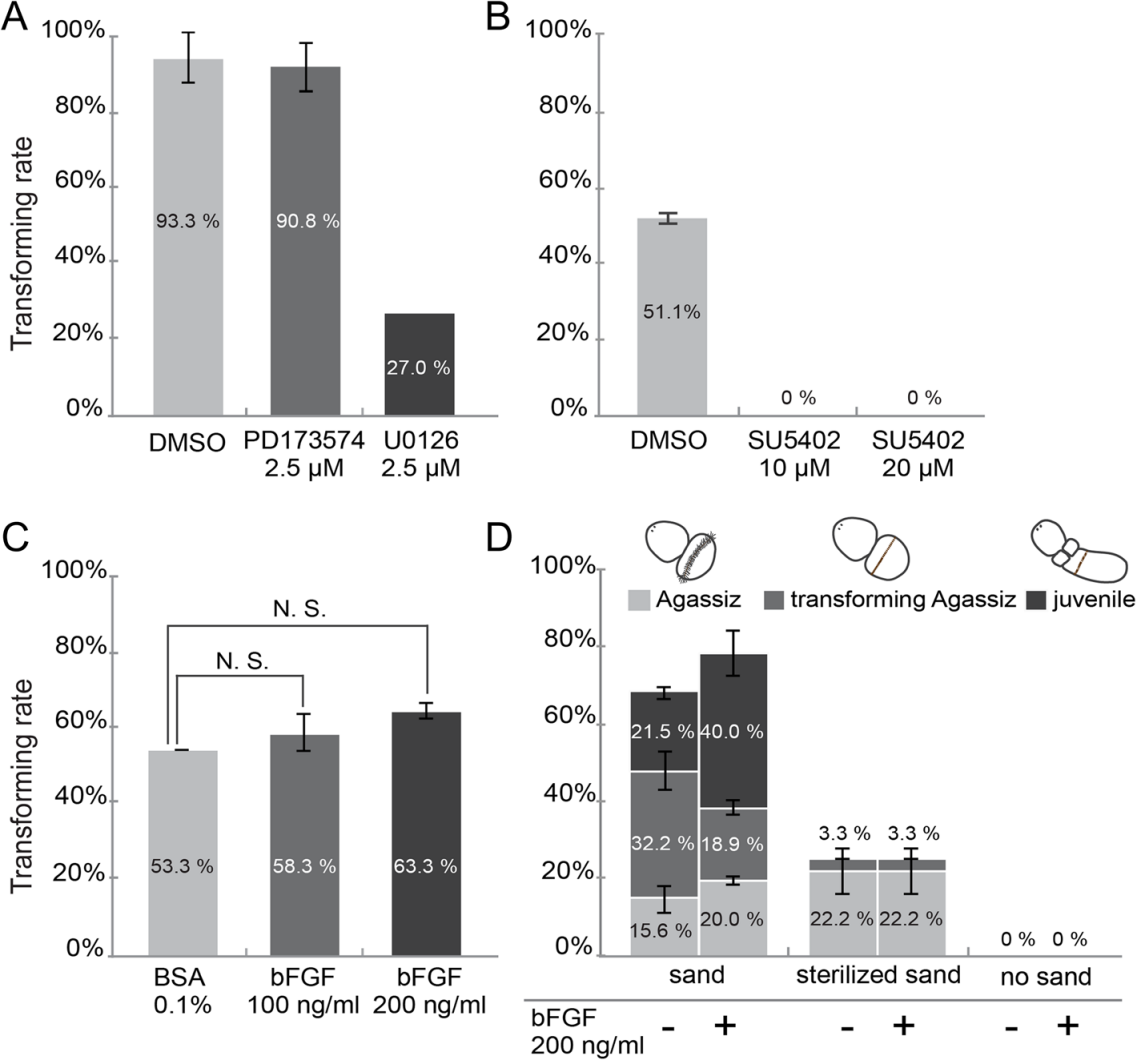
The effect of FGF signaling on sand-induced metamorphosis. **a-c** The transforming rate of the Spengel larvae after 2 days of incubation with the sand and PD173074, U0126 (**a**), SU5402 (**b**) or bFGF protein (**c**) at indicated concentrations. The transformation rate was calculated by dividing the total number of the Spengel larvae used in the experiment with the sum up number of the Spengel larvae transformed into the Agassiz, transforming Agassiz and juvenile stages. **d** The percentages of the Spengel larvae that transformed into the Agassiz, transforming Agassiz, or juvenile stages after 2 days of incubation with sand, sterilized sand, or without sand in the presence of bFGF (+) or BSA (−) protein are shown. Every experiment was repeated at least three times except the U0126 treatment, which was conducted only once. N.S: not statistically significant

Given that FGF signaling was required for muscle development during embryogenesis, we set out to test the potential role of FGF signaling in the production of muscle during metamorphosis. We first analyzed the expression of genes encoding FGF ligands, FGF receptors, and two myogenic Fox factors, *foxc* and *foxf*, in Spengel larvae and at the sand-induced Agassiz and juvenile stages. QPCR revealed that the transcript levels of these genes were all significantly increased at the Agassiz and juvenile stages, compared to the Spengel stage (Additional file 8: Figure S6; Additional file 7 for supplementary methods), suggesting their roles in this morphological transition. Inhibition of FGF signaling using the three different inhibitors, however, resulted in diverse results. Unlike PD173074, which had no detectable effect on sand-induced transformation, U0126 decreased the transformation rate substantially (Fig. 8a). Even more, SU5402 completely blocked sand-induced transformation and metamorphosis (Fig. 8b), and the treated larvae were morphologically indistinguishable with the untreated larva (Additional file 9: Figure S7A1–3). When FGF signaling was augmented by the addition of bFGF protein, the sand-induced transformation rate increased slightly, although the increase was not statistically significant (Fig. 8c; Student’s *t* test, *P* > 0.05). The morphology of the transforming larvae and juveniles upon bFGF treatments were also similar to the untreated individuals (Additional file 9: Figure S7B1-B6). We further analyzed the data by categorizing the stages as either Agassiz, transforming Agassiz or juvenile stages. By this analysis, we discovered that the percentage of larvae that had completed metamorphosis into juveniles was significantly increased when FGF signaling was elevated (Fig. 8d, first group; *X*^*2*^ test, *X*^*2*^ = 8.94, *df* = 3, 0.025 < *P* < 0.05). These results suggest that FGF signaling plays a positive role in promoting sand-induced metamorphosis. However, due to the conflicting results from the three FGF inhibitor treatments, it remains uncertain whether FGF signaling is required to trigger metamorphosis.

In a previous study, we showed that autoclaved sand is less efficient at inducing metamorphosis, suggesting that some unknown, sand-associated, heat and pressure-labile factors are required to stimulate this morphological transition [30]. To further examine the relationship between the environmental (sand) and physiological (FGF signaling) factors that participate in the induction of metamorphosis, we compared the transformation rates of larvae cultured with sand, sterilized sand, or no sand, in the presence or absence of exogenous bFGF protein. As observed previously [30], the rate of the sterilized sand-induced transformation was notably lower than that of regular sand. Furthermore, the addition of bFGF did not increase the rate of transformation in sterilized sand-treated larvae (Fig. 8d, the second group). In addition, no metamorphosis was observed when the larvae were cultured in seawater without sand, regardless of whether bFGF protein was present (Fig. 8d, the third group). Thus, physical contact with the sand appears to be indispensable for triggering metamorphosis, and FGF stimulation alone is not sufficient to induce the metamorphosis of *P. flava* in the absence of environmental chemical/biological stimulating factors.

## Discussion

### The conserved role of FGF8/17/18 in mesoderm induction

FGF homologs are widely distributed in bilaterians and cnidarians [57–59]. To date, at least 23 FGF ligands have been identified in tetrapods and are classified in seven subfamilies [60, 61], including FGF1/2, FGF3/7/10/22, FGF4/5/6, FGF8/17/18, FGF9/16/20, FGF 11/12/13/14 and FGF19/21/23. Among these subfamilies, FGF8/17/18 represents an ancestral family that may have emerged in the eumetazoan ancestor [58, 62]. The function of this family in mesoderm development has been demonstrated in multiple species. For example, a loss-of-function experiment in *Xenopus* demonstrated that *fgf8* is necessary for mesoderm formation [63]. Additionally, inhibition of both *fgf8* and *fgf24* (a paralogue of *fgf8*) in zebrafish blocks the formation of most posterior mesoderm [9]. Moreover, in ascidian embryos, the function of both FGF9/16/20 and FGF8/17/18 are essential for notochord formation [14]. The conserved function of FGF8/17/18 was also confirmed in the direct-developing hemichordate, *S. kowalevskii*, in which *fgf8/17/18* expressing cells are sufficient to induce mesoderm in adjacent cells [26]. In this study, we show that FGF signaling is crucial for mesoderm induction during embryogenesis in the indirect-developing hemichordate *P. flava*. The earliest FGF signaling in this species may also be mediated by FGF8/17/18, since it was one of the earliest FGF ligands to be produced (at the late blastula stage). The expression of *fgf8/17/18* in the apical ectoderm also coincided with that of its ortholog in *S. kowalevskii*, suggesting the conserved role of FGF8/17/18 in mediating embryonic mesoderm induction in hemichordates. However, there are significant differences in how the mesoderm-derived coeloms are formed in these two species. In *S. kowalevskii*, the coeloms are formed from pouches evaginated from the archenteron through a process called enterocoely and the embryo directly develops into the juvenile form within days [26]. In the indirect-developing *P. flava*, the protocoel, which is the anterior coelom, is also formed by enterocoely during embryogenesis. However, the formation of the paired posterior coeloms, the meso- and metacoels, is not by enterocoely but is through aggregation of the mesenchymal cells surrounding the digestive tract during the late larval stages, around two to 4 months after fertilization [30, 31]. Our study shows that the formation of the protocoel is FGF-dependent, although it remains unclear whether FGF signaling is required for the development of the posterior meso- and metacoels.

### The ancestral role of FGF signaling in myogenesis

The vertebrate embryonic mesoderm is divided into the axial, paraxial, intermediate and lateral domains. These domains exhibit different regulatory states that govern the formation of diverse cell types during development. Similarly, based on the expression patterns of *foxc*, *foxf*, *twist* and *myocardin*, we discovered that the *P. flava* embryonic mesoderm is partitioned into at least three domains, and two of which, the ventral and middle domains, are involved in myogenesis. In addition, we found that FGF signaling is required for the maintenance of *foxc* and *myocardin* expression in the ventral and middle domains and the formation of the two muscular structures, the pharyngeal muscle and the muscle string. The role of FGF signaling in myogenesis has also been suggested in *S. kowalevskii.* When the translation of *fgfr-b* or *fgf8/17/18* is inhibited by siRNAs, expression of muscle markers, such as muscle *Lim* protein and *myosin*, is greatly reduced [26]. In sea urchin embryos, the homologous myogenic factors are also expressed in discrete domains of coelomic pouches, which are paired mesodermal structures that form by enterocoely at the tip of the archenteron [52, 64]. Moreover, FGF signaling and the Fox factors in sea urchin embryos have also been shown to be a central part of the gene regulatory network controlling myogenesis [24]. The ontogeny and developmental mechanisms of the sea urchin coelomic pouches and the *P. flava* protocoel are thus similar, suggesting that these features are likely ancestral, at least in the common ancestor of the ambulacrarians.

### The reiterative use of FGF signaling during metamorphosis

In *P. flava*, metamorphosis takes only one to 2 days, but results in dramatic changes in both body plan and lifestyle. One intriguing finding in our study is that muscles are extensively generated during metamorphosis, coinciding with significant increases in the expression levels of the genes encoding FGF ligands, FGF receptors and myogenesis factors. Indeed, the dual observations that FGF signaling is required for embryonic myogenesis and that exogenous bFGF protein accelerated the transformation support the notion that FGF signaling plays a positive role in myogenesis during this morphological transition. However, the issue of whether FGF signaling is indispensable to this morphological transition was not unequivocally resolved in our study. This uncertainty is due to our variable results using two different FGF receptor inhibitors, PD173074 and SU5402. The discrepant results may have been generated because of two potential reasons. First, the concentrations of the two drugs that were used for metamorphosis experiments were optimized in embryos (2.5 μM for PD173074 and 20 μM for SU5402). The penetration efficiency of the drugs may be lower in the Spengel larva, which has a bigger body cavity than the smaller embryos. A lower penetration efficiency would reduce the effective drug dose, leading to a lack of biological efficacy at low doses, and this dampening of efficacy may have been especially relevant for PD173074, which was used at a relatively low concentration. Second, although SU5402 is strongly selective for human FGFRs, cell-free kinase assays have revealed that it is also a potent inhibitor of other tyrosine kinase receptors (e.g., FLT3 and JAK3) [65]. Therefore, the SU5402-mediated complete inhibition of sand-induced transformation may not be solely due to the inhibition of FGF signaling, but may also involve the inhibition of other tyrosine kinase receptors. Additionally, the mechanisms controlling metamorphosis are likely to be complex. To this point, our study revealed that metamorphosis in *P. flava* is induced by multiple factors. Both physical contact with the sand and some as yet unidentified, sand-associated chemical or biological factor(s) are required to stimulate metamorphosis. It remains unclear whether these environmental factors are directly responsible for the observed upregulation of FGF ligand and receptor genes during metamorphosis, and if so, how the upregulation might be mediated. Regardless of the necessity for FGF signaling in this process, our data clearly show that FGF can accelerate metamorphosis, possibly by activating myogenic factors and to trigger the formation of the muscle fibers during this time. This massive generation of muscle fibers during metamorphosis is necessary for lifestyle changes, in which the cilia-driven planktonic larva transforms into a muscle-driven benthic worm.

## Conclusions

In this study, we show that FGF signaling is required for mesoderm induction and muscle formation during embryogenesis of the hemichordate acorn worm *P. flava*. The extensive production of muscle and elevated expression of FGF ligand and receptor genes during metamorphosis strongly imply that FGF signaling is reiteratively used in mesoderm development during embryogenesis and metamorphosis in *P. flava.* Thus, the role of FGF signaling in muscle development is conserved at least in ambulacrarians and may have been present in the common ancestor of deuterostomes.

## Additional files

Additional file 1: **Table S1.** Accession numbers for genes/proteins used in this study. **Table S2.** Primers used for cloning. (DOCX 25 kb)

Additional file 2: **Figure S1.** Development of the mesodermal structures in *P. flava.* (A) The presumptive endomesoderm emerges as a thickened vegetal plate (yellow and red stripes) at the late blastula stage. (B) At the mid gastrula stage, the mesodermal cells (red) are specified at the tip of the archenteron (yellow). (C) At the late gastrula stage, the mesoderm develops into the protocoel that extends dorsally and forms a duct-like structure, the hydroporic canal (black arrow), which opens in the dorsal ectoderm to form a hydropore (black asterisk). (D) After hatching, the mesoderm of the tornaria larva further differentiates into the pharyngeal muscle (green arrow) and the muscle string (black arrowhead) that reaches to the anterior ectoderm. (E) At the Spengel larval stage, the protocoel is considerably enlarged, and two paired coeloms, the mesocoels (light purple) and metacoels (dark purple), form as two pairs of rings surrounding the stomach. (F) During metamorphosis, the protocoel forms a proboscis coelom at the Agassiz stage. (G) The transforming Agassiz has a more elongated posterior region, starts losing its cilia, and is incapable of swimming. (H) The juvenile has a typical tripartite body with an anterior proboscis, followed by a collar region and a trunk. The two black dots on the anterior ectoderm indicate the eye spots. Abbreviations: prob., proboscis; col., collar. (PNG 353 kb)

Additional file 3: Perturbations of FGF signaling after fertilization. Phenotypes of embryos at 43 hpf (A1-F1) and 73 hpf (A2-F2) after treatment with FGF signaling inhibitors (B1-D2) or bFGF protein (F1-F2) upon fertilization. Control embryos were treated with DMSO or 0.1% BSA. The concentrations of each drug or protein are indicated in each panel. All embryos are shown from a lateral view with the mouth on the left. All panels are shown in the same scale, according to the scale bar in A1. Abbreviations: me, mesoderm; en, endoderm. (PNG 2340 kb)

Additional file 4: Inhibitions of FGF signaling at various developmental stages. (A1-A2) Phenotypes of the control embryos (DMSO-treated) at 43 hpf and 73 hpf. (B1-D4) Phenotypes of the late gastrula stage *P. flava* that were treated with PD173074 (B1-B4), SU5402 (C1-C4) or U0126 (D1-D4) at different developmental stages (indicated by yellow circles on the left). (E1-H5) Tornaria larvae treated with SU5402 or U0126 at different developmental stages were observed and stained with Phalloidin (green). The larvae were counterstained with Hoechst 33,342 for nuclei (blue). The drugs and the concentrations used in the experiments are shown at the top of the panels, and the treatments were performed at the time points indicated by the yellow circles on the left. Abbreviations: ms, muscle string; pm, pharyngeal muscle; hc, hydroporic canal. (PNG 6751 kb)

Additional file 5: Expression patterns of the *snail*, *foxc*, *foxf*, *twist* and *myocardin* genes during embryogenesis. In situ hybridization for *snail* (A1-A7), *foxc* (B1-B7), *foxf* (C1-C7), *twist* (D1-D7) and *myocardin* (E1-E7) at different developmental stages indicated on the left. Embryos at 43 hpf and 73 hpf were viewed from the lateral side with mouth to the left. Embryos in the inlays of panels B6 and B7 were observed from the ventral side to show the expression of *foxc* in the posterior ciliary band. The expression patterns of each gene at the late gastrula and tornaria stages are delineated schematically below the corresponding panels. All panels are shown in the same scale, according to the scale bar in A1. (PNG 8481 kb)

Additional file 6: Phylogenetic and expression analyses of the two *MHC* genes identified in *P. flava.* **(A)** The amino acid sequences of the myosin head domains of the MHC proteins from 15 species were used to construct the phylogenetic tree. The three different types within the myosin superfamily, type I, II and V, were all well resolved. The two *P. flava* MHCs are grouped in type II (green branches), one within the skeletal/cardiac muscle MHC subgroup (*Pf*-stMHC) and the other in the smooth/non muscle subgroup (*Pf*-smMHC). Values at each node are Bootstrap inferences and the values lower than 50 are not shown. The scale bar indicates the substitutions per site. The abbreviations of the species names are: Amq, *Amphimedon queenslandica*; B, *Bovine*; Bb, *Branchiostoma belcheri*; Ce, *Caenorhabditis elegans*; Ci, *Ciona intestinalis*; Dm, *Drosophila melanogaster*; Hs, *Homo sapiens*; Mm, *Mus musculus*; Pf, *Ptychodera flava*; Pl, *Placopecten magellanicus*; Rn, *Rattus norvegicus*; Sk, *Saccoglossus kowalevskii*; Sc, *Saccharomyces cerevisiae*; Sch, *Schistosome mansoni*; Sp, *Strongylocentrotus purpuratus*. (B1-C2) In situ hybridization analyses of *P. flava stMHC* (B1-B2) and *smMHC* (C1-C2) in 43 hpf and 73 hpf embryos. The embryo in the inlay of B2 was viewed from the dorsal side to show the expression of *stMHC* in the muscle string. The embryo in the inlay of C1 was observed from the apical side. All panels are shown in the same scale, according to the scale bar in B1. The green arrow indicates the pharyngeal muscle and the black arrowheads denote the muscle string in B2. (PNG 1292 kb)

Additional file 7: Supplementary methods. (DOCX 77 kb)

Additional file 8: QPCR analyses of FGF ligands, FGF receptors, *foxc* and *foxf* during metamorphosis. The mRNA expression levels of genes encoding FGF ligands and receptors (A) and two myogenic factors, *foxc* (B) and *foxf* (C), were measured at the Spengel (gray), Agassiz (green) and juvenile (blue) stages. The gene names are given on the X-axis, and the relative expression levels normalized to 18S rRNA are shown by the bars. The numbers above each bar indicate the fold differences between gene expression levels in the indicated stages relative to the Spengel stage. (PNG 123 kb)

Additional file 9: Perturbations of FGF signaling during sand-induced metamorphosis. The morphology of a wild type Spengel larva (A1) and the Spengel larvae treated with 10 μM (A2) or 20 μM (A3) of SU5402. The images were taken 2 days after treatments. The morphology of individuals after cultured for 2 days with sand containing 0.1% BSA (B1–2), 100 ng/ml (B3–4) or 200 ng/ml bFGF protein (B5–6). The Spengel larvae transformed into either Agassiz (B1, B3, B5) or juveniles (B2, B4, B6). A1-A3 and B1-B6 are shown in two different scales, according to the scale bars in A1 and B1, respectively. (PNG 5404 kb)

## Abbreviations

bFGF: Basic-fibroblast growth factor
BSA: Bovine serum albumin
FGF: Fibroblast growth factor
FGFR: Fibroblast growth factor receptor
FSW: Filtered seawater
Hpf: Hour post fertilization
MEK: Mitogen-activated protein kinase kinase
ML: Maximum likelihood
PBST: Phosphate buffered saline containing 0.1% tween-20
QPCR: Quantitative polymerase chain reaction
SMART: Simple modular architecture research tool
SmMHC: Smooth muscle myosin heavy chain
StMHC: Striated muscle myosin heavy chain
