# Supplementary material for: Reiterative use of FGF signaling in mesoderm development during embryogenesis and metamorphosis in the hemichordate Ptychodera flava
Source: BMC Evol Biol. 2018 Aug 3;18:120. doi: 10.1186/s12862-018-1235-9 (PMC6091094; doi:10.1186/s12862-018-1235-9)
Supplement: Supplementary file 1 — Table S1. Accession numbers for genes/proteins used in this study. Table S2. Primers used for cloning. (DOCX 25 kb) [file 12862_2018_1235_MOESM1_ESM.docx]

**Table S1. Accession numbers for genes/proteins used in this study**

| **Clade** | **Species** | **Gene/protein Name** | **Accession Number** |
| --- | --- | --- | --- |
| *Hemichordata* | *Ptychodera flava* | *snail* | MG593808 |
|  |  | *foxc* | MG593809 |
|  |  | *foxf* | MG593810 |
|  |  | *myocardin* | MG593811 |
|  |  | *twist* | MG593812 |
|  |  | *stMHC* | MG593813 |
|  |  | *smMHC* | MG593814 |
|  | *Saccoglossus kowalevskii* | MHC | ALR88564.1 |
| *Saccharomycotina* | *Saccharomyces cerevisiae* | MYO 2 | NP_014971.1 |
|  |  | MYO 3 | AAB34124.1 |
|  |  | MYO 5 | NP_013827.1 |
| *Porifera* | *Amphimedon queenslandica* | stMHC | XP_003382837.1 |
|  |  | nmMHC | XP_003382828.1 |
| *Platyhelminthes* | *Schistosoma mansoni* | MHC | Q02456 |
|  |  | NMMHC-B | XM_018789588.1 |
| *Mullusca* | *Placopecten magellanicus* | MHC | Q26080 |
| *Nematoda* | *Caenorhabditis elegans* | HUM-2 | AAA97926.1 |
|  |  | NMY-2 | Q22869 |
| *Arthropoda* | *Drosophila melanogaster* | stMHC | NM_165181.3 |
|  |  | nmMHC | AAB09048.1 |
| *Urochordata* | *Ciona intestinalis* | MHC | XP_002119856.2 |
| *Cephalochordata* | *Branchiostoma belcheri* | MHC | XP_019620542.1 |
| *Echinodermata* | *Strongylocentrotus purpuratus* | MHC-X1 | XP_011682895.1 |
| *Vertebrata* | *Mus musculus* | MYH 1 | Q5SX40 |
|  |  | MYH 7 | NP_542766.1 |
|  |  | MYH 8 | P13542 |

| **Clade** | **Species** | **Gene/protein Name** | **Accession Number** |
| --- | --- | --- | --- |
| *Vertebrata* | *Mus musculus* | MYH 10 | Q61879 |
|  |  | MYH 11 | O08638 |
|  |  | MYH 14 | Q6URW6 |
|  |  | myosin I | AAA39800.1 |
|  | *Rattus norvegicus* | MYR 6 | XP_008770307.1 |
|  | *Bos taurus* | BBMI | P10568.1 |
|  | *Homo sapiens* | MYH 2 | Q9UKX2.1 |
|  |  | MYH 3 | P11055 |
|  |  | MYH 6 | AAI32668.1 |
|  |  | MYH 7 | P12883.5 |
|  |  | MYH 11 | AAS98910.1 |

**Table S1. Continued**

**Table S2. Primers used for cloning**

| **Gene Name** | **primer direction** | **Sequence** |
| --- | --- | --- |
| *snail* | F | CGCATGTCATTGCTTACC |
|  | R | GAATAAGTACTGGGGTCGCATCG |
| *foxc* | F | GTGGCAATAGCGAACTTAACTTGAG |
|  | R | CACAAAGAAAGTAGGGCTCGACT |
| *foxf* | F | CCCCGACCAAACGACTGACTCTC |
|  | R | GCAAACCCAGAGCATGAGTGACC |
| *myocardin* | F | GTGAAGCAACTTGAAATGACAAC |
|  | R | GTGAAAATAGAACCCAATGAATCG |
| *twist* | F | GGATGTGATTCAAGCGATGCAC |
|  | R | CTCACGAAAGACTCAGTTACGCT |
| *stMHC* | F | CCAGAAGAACATAAAGAAACTGCAA |
|  | R | AGGTGGCTATTTAACAAGAAC |
| *smMHC* | F | CTGGATGAAGCGGAGGAAGAAG |
|  | R | GGACATTAGAGTTGCAGGTATTGGT |

Abbreviations: F, forward; R, reverse.
