# Supplementary figures and images for: Reiterative use of FGF signaling in mesoderm development during embryogenesis and metamorphosis in the hemichordate Ptychodera flava
Source: BMC Evol Biol. 2018 Aug 3;18:120. doi: 10.1186/s12862-018-1235-9 (PMC6091094; doi:10.1186/s12862-018-1235-9)

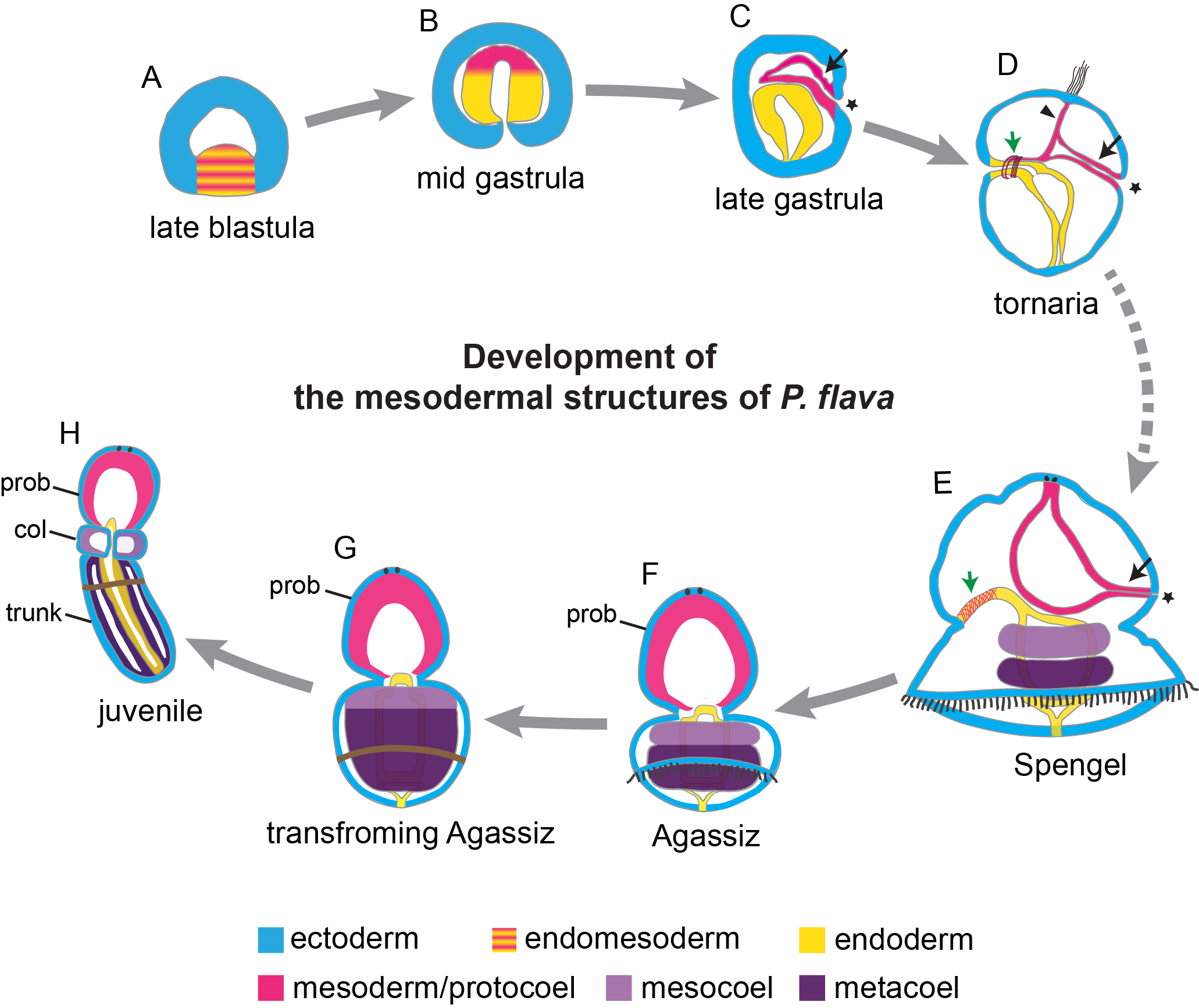

Supplement: Supplementary file 2 — Figure S1. Development of the mesodermal structures in P. flava. (A) The presumptive endomesoderm emerges as a thickened vegetal plate (yellow and red stripes) at the late blastula stage. (B) At the mid gastrula stage, the mesodermal cells (red) are specified at the tip of the archenteron (yellow). (C) At the late gastrula stage, the mesoderm develops into the protocoel that extends dorsally and forms a duct-like structure, the hydroporic canal (black arrow), which opens in the dorsal ectoderm to form a hydropore (black asterisk). (D) After hatching, the mesoderm of the tornaria larva further differentiates into the pharyngeal muscle (green arrow) and the muscle string (black arrowhead) that reaches to the anterior ectoderm. (E) At the Spengel larval stage, the protocoel is considerably enlarged, and two paired coeloms, the mesocoels (light purple) and metacoels (dark purple), form as two pairs of rings surrounding the stomach. (F) During metamorphosis, the protocoel forms a proboscis coelom at the Agassiz stage. (G) The transforming Agassiz has a more elongated posterior region, starts losing its cilia, and is incapable of swimming. (H) The juvenile has a typical tripartite body with an anterior proboscis, followed by a collar region and a trunk. The two black dots on the anterior ectoderm indicate the eye spots. Abbreviations: prob., proboscis; col., collar. (PNG 353 kb) [file 12862_2018_1235_MOESM2_ESM.png]

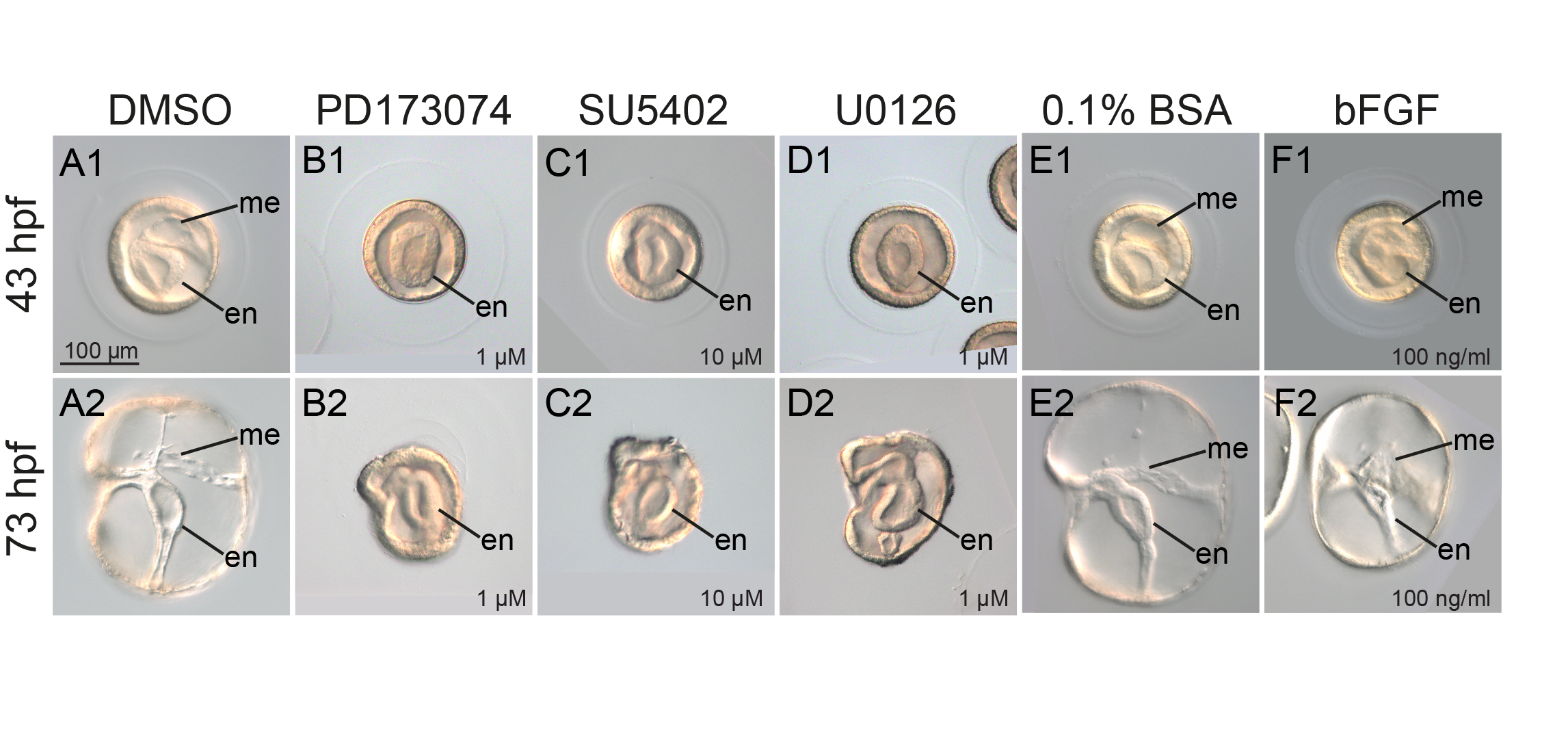

Supplement: Supplementary file 3 — Perturbations of FGF signaling after fertilization. Phenotypes of embryos at 43 hpf (A1-F1) and 73 hpf (A2-F2) after treatment with FGF signaling inhibitors (B1-D2) or bFGF protein (F1-F2) upon fertilization. Control embryos were treated with DMSO or 0.1% BSA. The concentrations of each drug or protein are indicated in each panel. All embryos are shown from a lateral view with the mouth on the left. All panels are shown in the same scale, according to the scale bar in A1. Abbreviations: me, mesoderm; en, endoderm. (PNG 2340 kb) [file 12862_2018_1235_MOESM3_ESM.png]

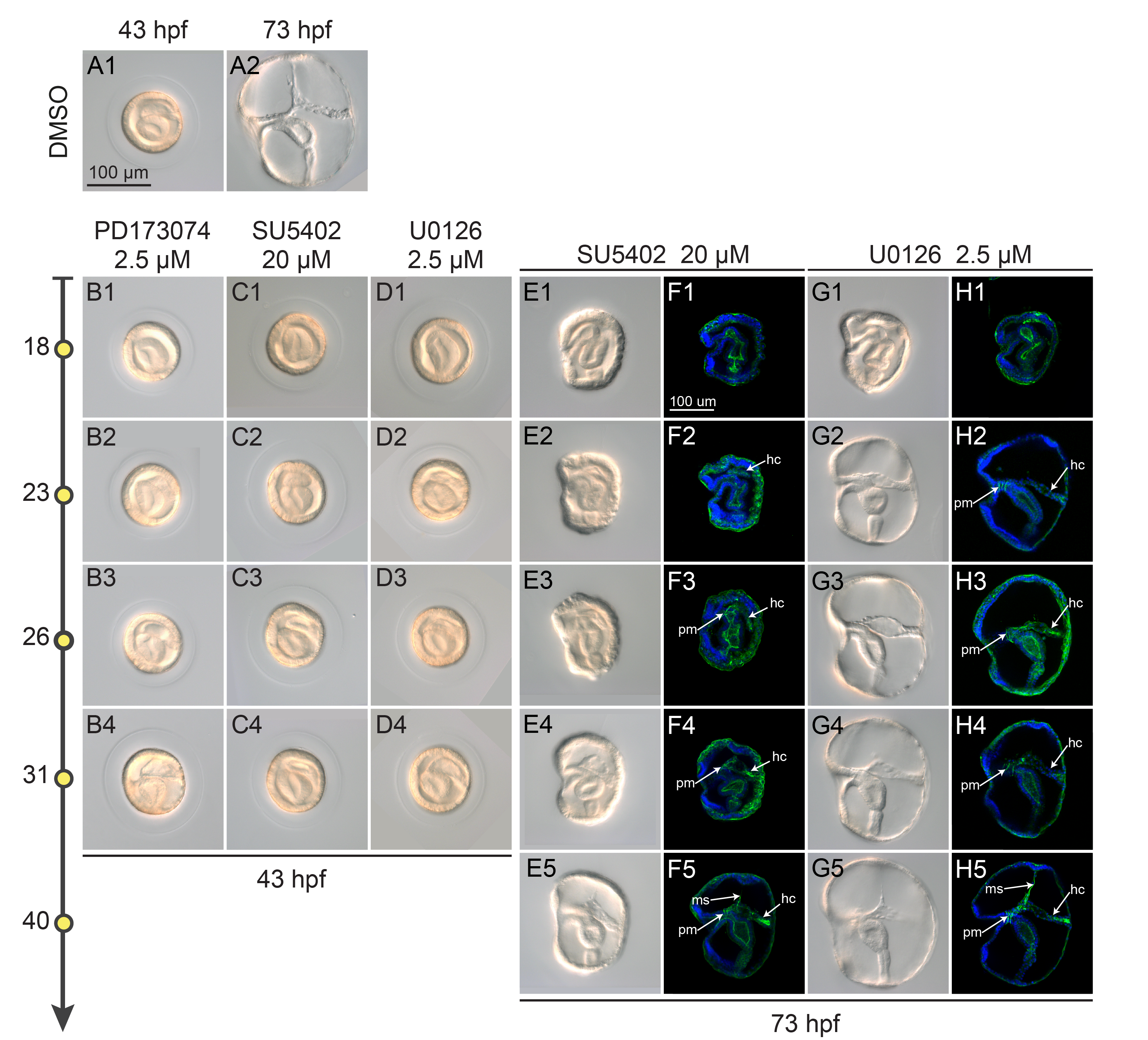

Supplement: Supplementary file 4 — Inhibitions of FGF signaling at various developmental stages. (A1-A2) Phenotypes of the control embryos (DMSO-treated) at 43 hpf and 73 hpf. (B1-D4) Phenotypes of the late gastrula stage P. flava that were treated with PD173074 (B1-B4), SU5402 (C1-C4) or U0126 (D1-D4) at different developmental stages (indicated by yellow circles on the left). (E1-H5) Tornaria larvae treated with SU5402 or U0126 at different developmental stages were observed and stained with Phalloidin (green). The larvae were counterstained with Hoechst 33,342 for nuclei (blue). The drugs and the concentrations used in the experiments are shown at the top of the panels, and the treatments were performed at the time points indicated by the yellow circles on the left. Abbreviations: ms, muscle string; pm, pharyngeal muscle; hc, hydroporic canal. (PNG 6751 kb) [file 12862_2018_1235_MOESM4_ESM.png]

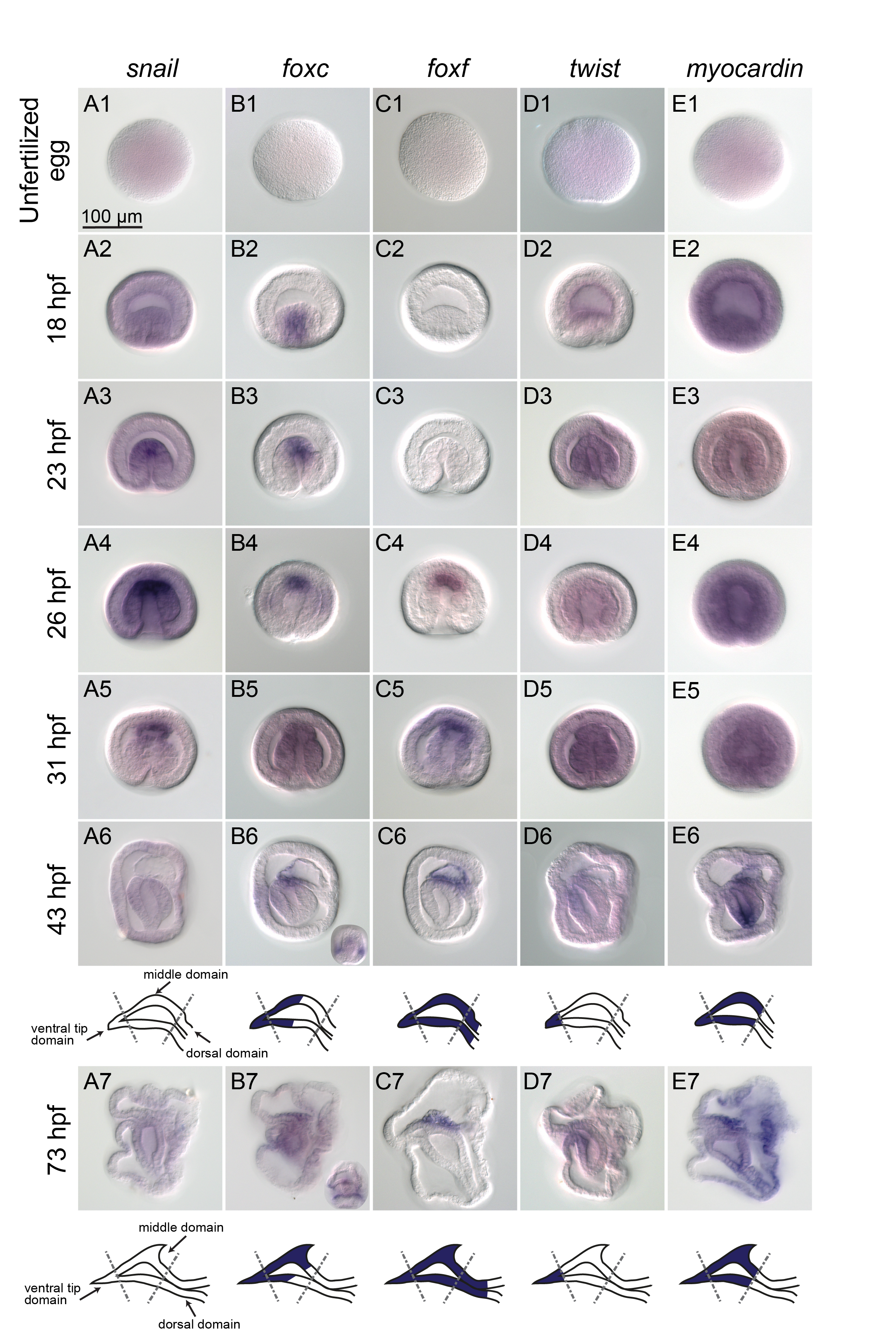

Supplement: Supplementary file 5 — Expression patterns of the snail, foxc, foxf, twist and myocardin genes during embryogenesis. In situ hybridization for snail (A1-A7), foxc (B1-B7), foxf (C1-C7), twist (D1-D7) and myocardin (E1-E7) at different developmental stages indicated on the left. Embryos at 43 hpf and 73 hpf were viewed from the lateral side with mouth to the left. Embryos in the inlays of panels B6 and B7 were observed from the ventral side to show the expression of foxc in the posterior ciliary band. The expression patterns of each gene at the late gastrula and tornaria stages are delineated schematically below the corresponding panels. All panels are shown in the same scale, according to the scale bar in A1. (PNG 8481 kb) [file 12862_2018_1235_MOESM5_ESM.png]

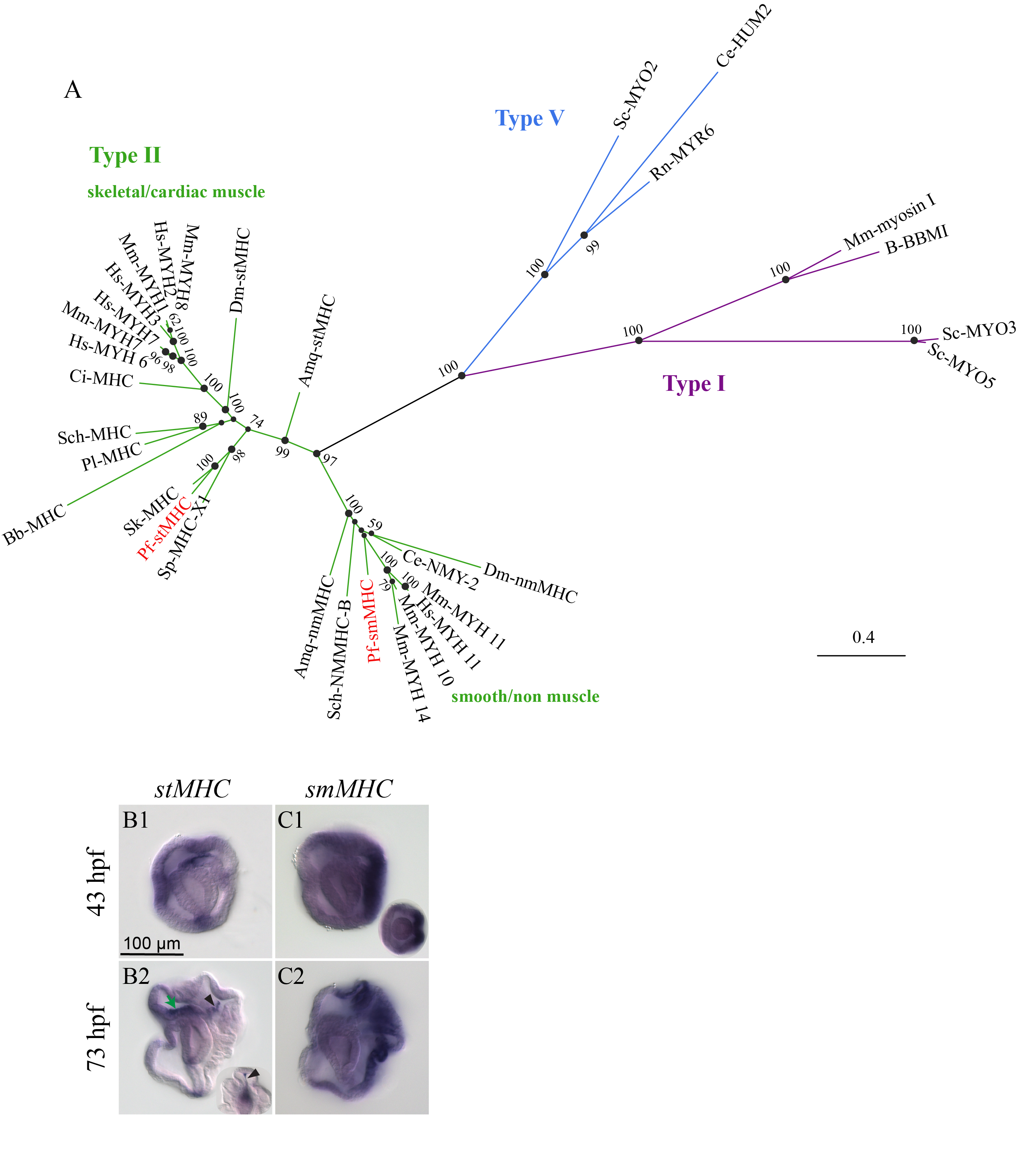

Supplement: Supplementary file 6 — Phylogenetic and expression analyses of the two MHC genes identified in P. flava. (A) The amino acid sequences of the myosin head domains of the MHC proteins from 15 species were used to construct the phylogenetic tree. The three different types within the myosin superfamily, type I, II and V, were all well resolved. The two P. flava MHCs are grouped in type II (green branches), one within the skeletal/cardiac muscle MHC subgroup (Pf-stMHC) and the other in the smooth/non muscle subgroup (Pf-smMHC). Values at each node are Bootstrap inferences and the values lower than 50 are not shown. The scale bar indicates the substitutions per site. The abbreviations of the species names are: Amq, Amphimedon queenslandica; B, Bovine; Bb, Branchiostoma belcheri; Ce, Caenorhabditis elegans; Ci, Ciona intestinalis; Dm, Drosophila melanogaster; Hs, Homo sapiens; Mm, Mus musculus; Pf, Ptychodera flava; Pl, Placopecten magellanicus; Rn, Rattus norvegicus; Sk, Saccoglossus kowalevskii; Sc, Saccharomyces cerevisiae; Sch, Schistosome mansoni; Sp, Strongylocentrotus purpuratus. (B1-C2) In situ hybridization analyses of P. flava stMHC (B1-B2) and smMHC (C1-C2) in 43 hpf and 73 hpf embryos. The embryo in the inlay of B2 was viewed from the dorsal side to show the expression of stMHC in the muscle string. The embryo in the inlay of C1 was observed from the apical side. All panels are shown in the same scale, according to the scale bar in B1. The green arrow indicates the pharyngeal muscle and the black arrowheads denote the muscle string in B2. (PNG 1292 kb) [file 12862_2018_1235_MOESM6_ESM.png]

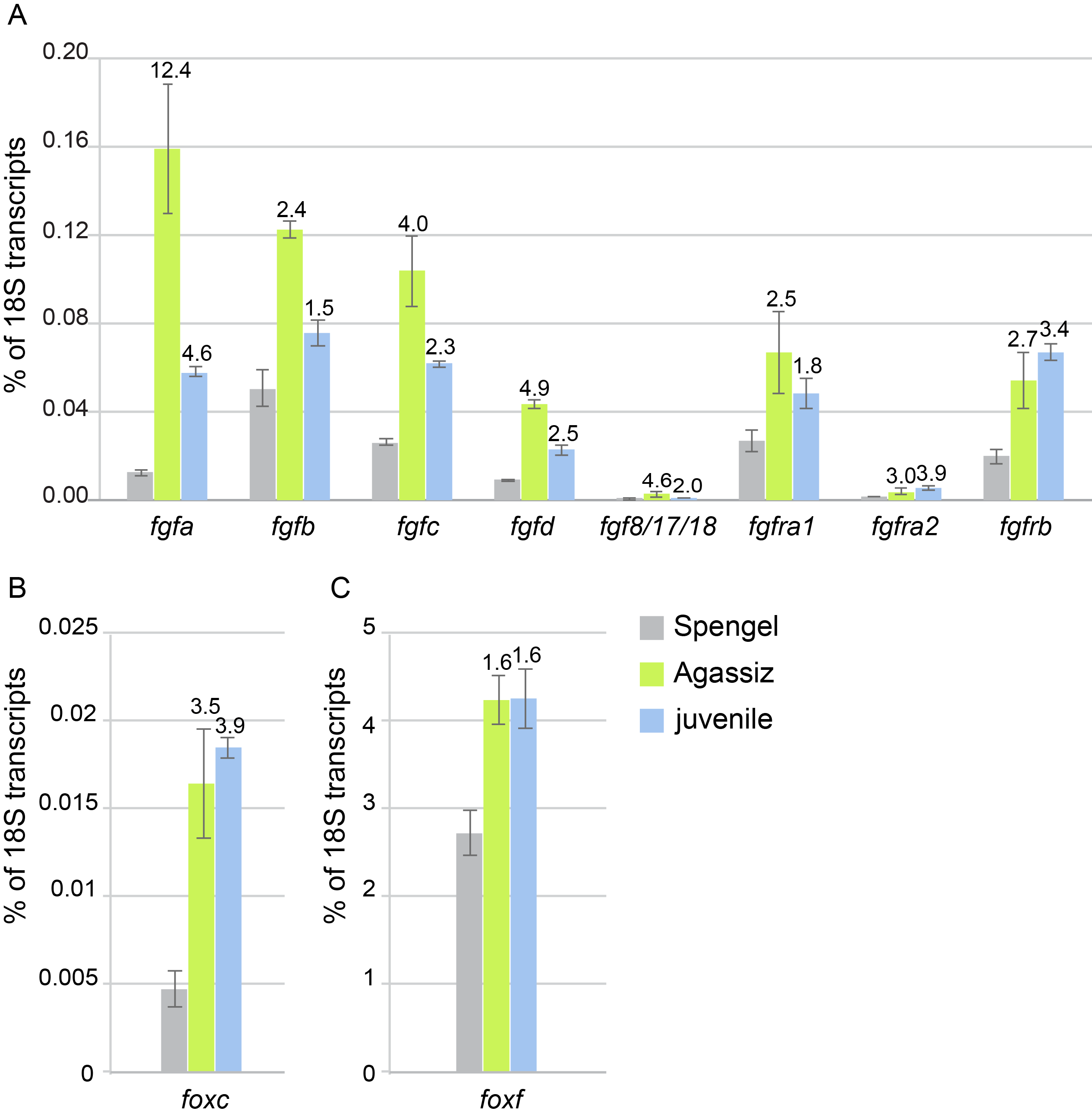

Supplement: Supplementary file 8 — QPCR analyses of FGF ligands, FGF receptors, foxc and foxf during metamorphosis. The mRNA expression levels of genes encoding FGF ligands and receptors (A) and two myogenic factors, foxc (B) and foxf (C), were measured at the Spengel (gray), Agassiz (green) and juvenile (blue) stages. The gene names are given on the X-axis, and the relative expression levels normalized to 18S rRNA are shown by the bars. The numbers above each bar indicate the fold differences between gene expression levels in the indicated stages relative to the Spengel stage. (PNG 123 kb) [file 12862_2018_1235_MOESM8_ESM.png]

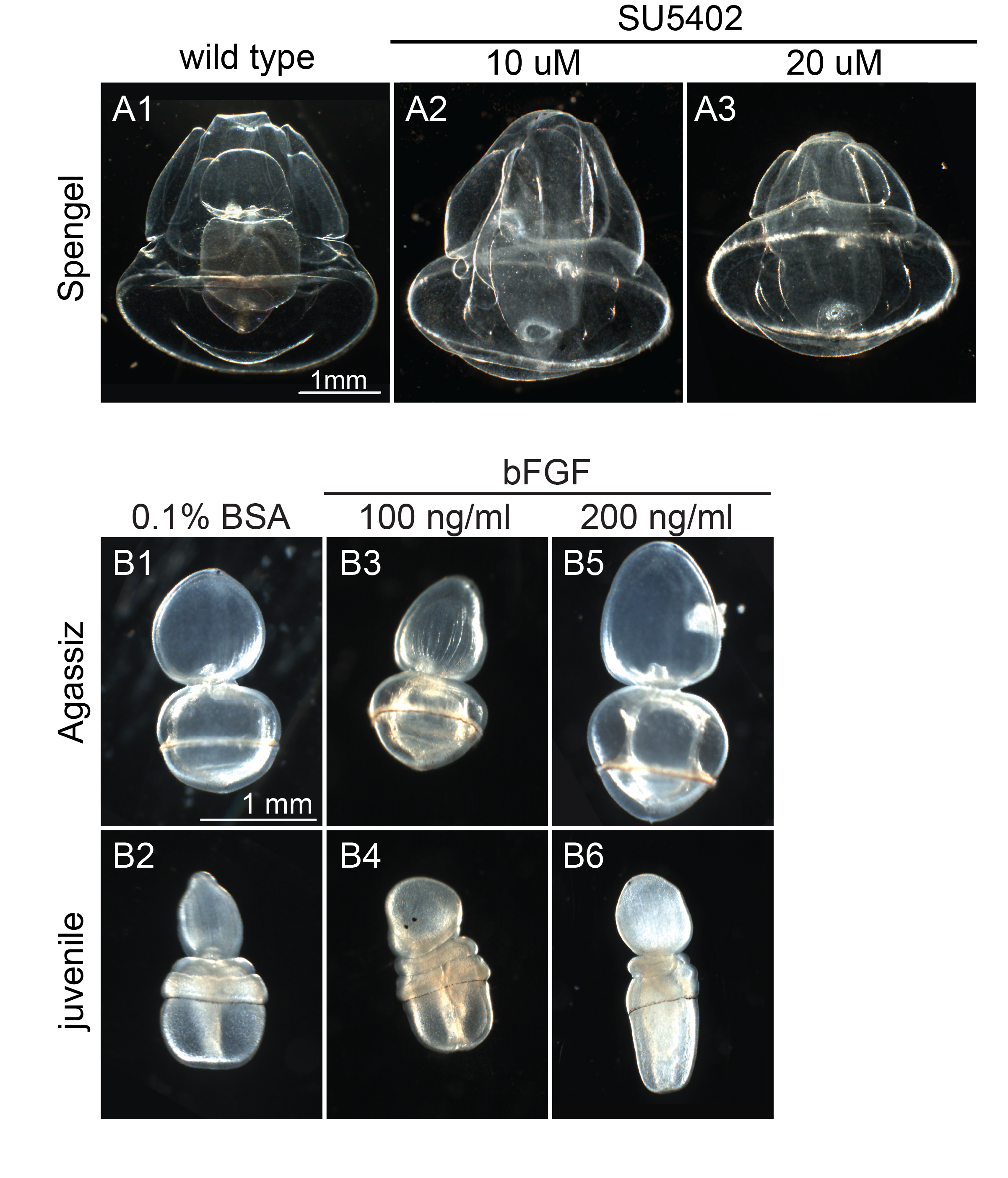

Supplement: Supplementary file 9 — Perturbations of FGF signaling during sand-induced metamorphosis. The morphology of a wild type Spengel larva (A1) and the Spengel larvae treated with 10 μM (A2) or 20 μM (A3) of SU5402. The images were taken 2 days after treatments. The morphology of individuals after cultured for 2 days with sand containing 0.1% BSA (B1–2), 100 ng/ml (B3–4) or 200 ng/ml bFGF protein (B5–6). The Spengel larvae transformed into either Agassiz (B1, B3, B5) or juveniles (B2, B4, B6). A1-A3 and B1-B6 are shown in two different scales, according to the scale bars in A1 and B1, respectively. (PNG 5404 kb) [file 12862_2018_1235_MOESM9_ESM.png]
