# Supplementary material for: Reiterative use of FGF signaling in mesoderm development during embryogenesis and metamorphosis in the hemichordate Ptychodera flava
Source: BMC Evol Biol. 2018 Aug 3;18:120. doi: 10.1186/s12862-018-1235-9 (PMC6091094; doi:10.1186/s12862-018-1235-9)
Supplement: Supplementary file 7 — Supplementary methods. (DOCX 77 kb) [file 12862_2018_1235_MOESM7_ESM.docx]

**Supplementary methods**

**Quantitative polymerase chain reaction (QPCR)**

Total RNA was extracted using TRIzol reagent (Invitrogen) from organisms at several developmental stages, including the unfertilized eggs, embryos at 12, 17, 25, 32, 43 and 64 hpf, and the Spengel, Agassiz and juvenile stages. The extracted RNA was further purified with an RNeasy Micro kit (Qiagen, Chatsworth, CA, USA). Up to 1 μg of total RNA from each stage was reverse transcribed individually for QPCR. The levels of 18S rRNA were used for normalizing samples.

**Phylogenetic analysis**

The protein sequences used to construct the phylogenetic tree were obtained from the GenBank (see Table S1 for the accession numbers). The *P. flava* stMHC and smMHC sequences were deduced from the cDNA sequences cloned in this study. The myosin head domains of the MHC proteins were predicted using the online SMART software [1, 2] and aligned with ClustalW [3]. Gaps in alignment were removed manually. Maximum Likelihood (ML) analysis was inferred using RAxML-HPC BlackBox 8.2.10 [4] with the LG model (CIPRES Science Gateway v. 3.3). Values at each node were generated by 1000 bootstrap iterations.

**References**

1. Letunic I, Doerks T, Bork P. SMART: recent updates, new developments and status in 2015. Nucleic Acids Res 2015;43:D257-60.

2. Schultz J, Milpetz F, Bork P, Ponting CP. SMART, a simple modular architecture research tool: identification of signaling domains. Proc Natl Acad Sci U S A 1998;95:5857-64.

3. Thompson JD, Higgins DG, Gibson TJ. CLUSTAL W: improving the sensitivity of progressive multiple sequence alignment through sequence weighting, position-specific gap penalties and weight matrix choice. Nucleic Acids Res 1994;22:4673-80.

4. Stamatakis A. RAxML version 8: a tool for phylogenetic analysis and post-analysis of large phylogenies. Bioinformatics 2014;30:1312-3.
